# Extra-viral DNA in adeno-associated viral vector preparations induces TLR9-dependent innate immune responses in human plasmacytoid dendritic cells

**DOI:** 10.1038/s41598-023-28830-7

**Published:** 2023-02-02

**Authors:** Kirsten Bucher, Eduardo Rodríguez-Bocanegra, Bernd Wissinger, Torsten Strasser, Simon J. Clark, Andreas L. Birkenfeld, Dorothea Siegel-Axel, M. Dominik Fischer

**Affiliations:** 1 University Eye Hospital, Centre for Ophthalmology, University Hospital Tübingen, Tübingen, Germany; 2 Institute for Ophthalmic Research, Centre for Ophthalmology, University Hospital Tübingen, University of Tübingen, Elfriede-Aulhorn-Strasse 7, 72076 Tübingen, Germany; 3 Lydia Becker Institute of Immunology and Inflammation, Faculty of Biology, Medicine and Health, University of Manchester, Manchester, UK; 4 Institute of Diabetes Research and Metabolic Diseases (IDM) of the Helmholtz Center Munich, Tübingen, Germany; 5 German Center for Diabetes Research (DZD), Neuherberg, Germany; 6 Division of Endocrinology, Diabetology and Nephrology, Department of Internal Medicine IV, University Hospital of Tübingen, Tübingen, Germany; 7 Oxford Eye Hospital, Oxford University NHS Foundation Trust, Oxford, UK; 8 Nuffield Laboratory of Ophthalmology, Department of Clinical Neurosciences, University of Oxford, Oxford, UK

**Keywords:** Immunology, Antimicrobial responses, Cytokines, Innate immunity, Medical research, Drug development

## Abstract

Adeno-associated viral (AAV) vector suspensions produced in either human derived HEK cells or in *Spodoptera frugiperda* (*Sf9*) insect cells differ in terms of residual host cell components as well as species-specific post-translational modifications displayed on the AAV capsid proteins. Here we analysed the impact of these differences on the immunogenic properties of the vector. We stimulated human plasmacytoid dendritic cells with various lots of HEK cell-produced and *Sf9* cell-produced AAV-CMV-eGFP vectors derived from different manufacturers. We found that AAV8-CMV-eGFP as well as AAV2-CMV-eGFP vectors induced lot-specific but not production platform-specific or manufacturer-specific inflammatory cytokine responses. These could be reduced or abolished by blocking toll-like receptor 9 signalling or by enzymatically reducing DNA in the vector lots using DNase. Successful HEK cell transduction by DNase-treated AAV lots and DNA analyses demonstrated that DNase did not affect the integrity of the vector but degraded extra-viral DNA. We conclude that both HEK- and *Sf9*-cell derived AAV preparations can contain immunogenic extra-viral DNA components which can trigger lot-specific inflammatory immune responses. This suggests that improved strategies to remove extra-viral DNA impurities may be instrumental in reducing the immunogenic properties of AAV vector preparations.

## Introduction

The last decade has witnessed an enormous interest in the development of new recombinant adeno-associated virus (AAV)-based strategies for both basic research and clinical applications^1–3^. The major reason lies in the fact that AAV vectors have been extremely versatile tools in the field of gene therapy given their ability to efficiently and safely deliver therapeutic genes to target tissues^4^. However, a growing number of investigators independently report findings suggesting local and systemic immune responses after delivery of AAV in pre-clinical and clinical studies^5–8^.

AAV vectors have been shown to activate innate immune pattern recognition receptors such as toll-like receptor (TLR)2 and TLR9 resulting in the release of inflammatory cytokines and type I interferons (IFN)^9,10^. Immunogenic components of AAV that might stimulate these responses include capsid proteins and vector genome^9,10^. Immune responses after AAV application might also be triggered by impurities in the vector suspension^5,11^. These impurities have been defined as any component present in the purified AAV vector suspension other than the desired product^12^ and result from the production process of the vector. Two of the major methods of producing clinical recombinant AAV vectors involve the transfection of plasmid DNA into HEK cells and the infection of *Spodoptera frugiperda* (*Sf9*) insect cells with bacoluvirus^13^. Accordingly, potentially immunogenic impurities contained in AAV vector suspensions could include endotoxins, cell culture medium components, reagents that are used for AAV purification, proteins and DNA derived from host cells and residual baculoviral DNA or plasmid DNA^5^. Additional factors that could influence the immunogenicity of AAV vector suspensions are post-translational modifications (PTMs) imprinted to the capsid by the different vector production platforms^5^.

Importantly, HEK cell-derived and *Sf9* cell-derived AAV vectors differ greatly in terms of their PTMs, their residual host cell protein impurities^11^, and potentially also in their DNA impurities (HEK cell DNA vs.* Sf9* cell DNA as well as residual plasmid DNA vs. baculoviral DNA)^14^. Plasmacytoid dendritic cells (pCDs) are a specialized innate immune cell type that secretes large amounts of type I IFN and pro-inflammatory cytokines upon viral infections^9,15^ and play a major role in the sensing of AAV vectors^9^. Accordingly, to analyse whether the differences between HEK cell-produced and Sf9-cell-produced AAV vectors result in differences in their immunogenic properties, we stimulated human pDCs with different lots of the same AAV vector obtained from the two production systems and different manufacturers. We found that half of the vector lots examined elicited lot-specific pro-inflammatory immune responses that were neither related to the vector production system nor the manufacturer. These responses were mediated by TLR9 signalling and susceptible to treatment of the vector lots with DNase. Successful HEK cell transduction by both untreated and DNase-treated AAV vector lots and DNA analyses of AAV preparations suggested that DNase did not affect AAV particle integrity but rather targeted non-encapsulated extra-viral DNA. Collectively, this suggests that AAV vector preparations can contain non-encapsulated extra-viral DNA which can influence immunogenic properties of AAV vector preparations in human pDCs.

## Results

### AAV induces lot-specific innate immune responses in human pDCs

Studies have demonstrated that HEK cell-derived, and *Sf9* cell-derived, AAV vectors differ in terms of their PTMs and impurities contained in the viral suspensions^11^. We hypothesized that these factors could result in differences in the immunogenic properties between HEK cell- and *Sf9* cell-derived vector lots. To test this hypothesis, we analysed a total of eight AAV serotype 8 (AAV8) vector lots containing the identical DNA sequence of cytomegalovirus promoter (CMV) and the identical transgene for the enhanced green fluorescence protein (eGFP) (AAV8-CMV-eGFP; five HEK- and three *Sf9* cell-derived lots) and four AAV2-CMV-eGFP lots (two HEK- and two *Sf9* cell-derived lots) from three different manufacturers [manufacturer A; Viral Vector Core Facility of the University of Iowa (Iowa, USA), manufacturer B; Virovek (CA, USA) and manufacturer C; Vigene Biosciences (MD, USA)]. In order to maximize similarities among the lots, the seven AAV8 lots from manufacturer A and manufacturer B (Table 1) were produced using the same original plasmid. Additionally, a droplet digital PCR (ddPCR) re-titration of the vector genomes (vg) of the AAV vector lots was performed in a side-by-side measurement using two different targets: one within the CMV sequence and the other within the eGFP sequence of the vectors. Results obtained by the quantification of the CMV target were used for the titration of the AAV vector lots in the following experiments (Table 1).

**Table 1:** Different lots of AAV8 and AAV2 viral vectors used in this study.

AAV	Lot number	CMV target (vg/ml)	eGFP target (vg/ml)	Production platform	Manufacturer
AAV8-CMV-eGFP	A-HEK-1	6.53 E+12	6.69 E+13	HEK293 cells	University of Iowa
	A-HEK-2	4.61 E+12	4.57 E+12	HEK293 cells	University of Iowa
	A-HEK-3	5.88 E+12	5.97 E+12	HEK293 cells	University of Iowa
	A-Sf9-1	4.45 E+13	5.09 E+13	*Sf9* cells	University of Iowa
	A-Sf9-2	1.15 E+14	1.31 E+14	*Sf9* cells	University of Iowa
	B-HEK-1	5.24 E+12	5.83 E+12	HEK293 cells	Virovek
	B-Sf9-1	2.50 E+13	2.84 E+13	*Sf9* cells	Virovek
	C-HEK-1	3.63 E+13	3.69 E+13	HEK293 cells	Vigene
AAV2-CMV-eGFP	B-Sf9-1	2.47 E+13	2.74E+13	*Sf9* cells	Virovek
	B-Sf9-2	1.96 E+13	2.06E+13	*Sf9* cells	Virovek
	C-HEK-1	9.59 E+12	1.23E+13	HEK293 cells	Vigene
	C-HEK-2	9.73 E+12	1.12E+13	HEK293 cells	Vigene

**Figure 1:**
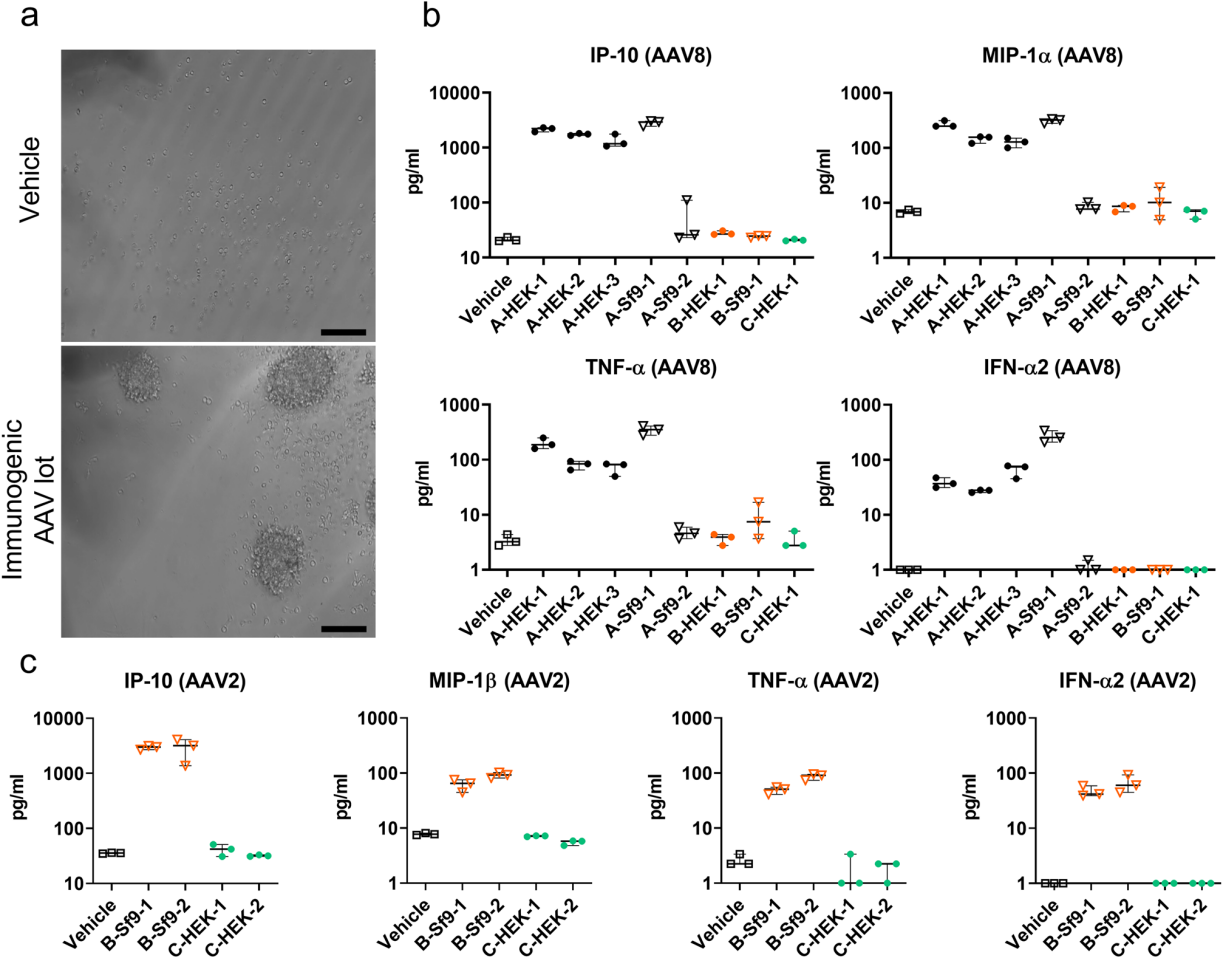
Induction of AAV vector lot-specific immune responses in human pDCs. Human pDCs were stimulated with different lots of AAV8-CMV-eGFP and AAV2-CMV-eGFP (MOI: 1:1 × 10^6^ vg) for 18 h (**a**) Representative bright field images of pDCs stimulated with vehicle control (upper image) or an immunogenic AAV vector lot (lower image). Scale bar is 100 μm. (**b**) Cytokine release of IP-10, MIP-1β, TNF-α and IFN-α2 by AAV8-stimulated pDCs. (**C**) Cytokine release by AAV2-stimulated pDCs. Representative plots of one of three to four independent experiments. Since in the IFN-α2 measurements (**b**,**c**) and TNF-α measurement (**c**) some values fell below the assay range, the constant 1 was added to all measured IFN-α2 and TNF-α values for presentation in a semi-logarithmic plot. Shown are medians and interquartile ranges. In the labels of the individual vector lots the three manufacturers are represented by the letters A, B, C; HEK-cell derived and Sf9-cell derived vectors are indicated by "HEK and "Sf9" and corresponding lots of the same manufacturer and the same production system are numbered “1, 2, 3”. Circle: HEK-derived vector lot; triangle: *Sf9*-derived vector lot; black: vector lot from manufacturer A; orange: vector lot from manufacturer B; green: vector lot from manufacturer C.

pDCs are specialized viral sensors that massively produce type I IFNs upon viral infection^15^, including AAV vectors^9^. To investigate whether HEK-cell produced and *Sf9*-cell produced AAV vectors differ in their capacity to elicit innate immune responses in immunocompetent cells, we stimulated human pDCs with the above listed AAV vector lots. To this end pDCs were purified from peripheral blood mononuclear cells (PBMCs) of individual healthy human donors by negative selection using magnetic activated cell sorting (MACS). Flow cytometry analysis confirmed a purity of the isolated pDCs of over 90% (Fig. S1). Then, pDCs of an individual donor were seeded and stimulated with AAV8 and AAV2 vector lots at an MOI of 1:1 × 10^6^ vg/cell for 18 h. The MOI of 1:10^6^ vg/cell applied to a total of 12.500 cells in 50 ul/well translates into a titre of 2.5 × 10^11^ vg/ml. We used this titre because it is within the range of what is applied in retinal gene therapy studies in humans (e.g. 1 × 10^12^ vg/ml^16^ or 4 × 10^11^–1.3 × 10^12^ vg/ml^17^) and non-human primates (e.g. 5 × 10^11^–5 × 10^12^ vg/ml^6^). Stimulation with vehicle served as control. Incubation with AAV8 and AAV2 vectors lots did not result in any detectable transgene expression in pDCs. However, four of the eight AAV8 lots (lots A-HEK-1, A-HEK-2, A-HEK-3, A-Sf9-1) and two of the four AAV2 lots (lots B-Sf9-1, B-Sf9-2) induced reactive cell proliferation in the stimulated pDCs (Fig. 1a). This was accompanied by a release of pro-inflammatory cytokines (IP-10, MIP-1β and TNF-α) and type I IFN (IFN-α). Conversely, neither cell proliferation nor cytokine release was induced by the remaining AAV8 (lots A-Sf9-1, B-HEK-1, B-Sf9-1, C-HEK-1) and AAV2 lots (lots C-HEK-1, C-HEK-2). These results were confirmed in three to four independent experiments each performed with the cells of one individual donor (Fig. 1a–c and Table S1). Lot-specific differences in the cytokine concentrations in these independent experiments were statistically analysed using linear mixed effect model and post hoc Dunnett’s test (Q = 2.6) by comparing the least-square means of the different AAV vector lots with the vehicle control (Tables S2 and S3). This demonstrated statistical significant differences in the cytokine responses between “immunogenic” AAV vector lots (AAV8 lots A-HEK-1, A-HEK-2, A-HEK-3, A-Sf9-1, or AAV2 lots B-Sf9-1, B-Sf9-2 respectively) and control but no significant differences between “non-immunogenic” AAV vector lots (AAV8 lots A-Sf9-1, B-HEK-1, B-Sf9-1, C-HEK-1 or AAV2 lots C-HEK-1, C-HEK-2 respectively) and control (Table S3). The concentrations of the rest of the measured cytokines included in the multiplex assay either were below the assay range (i.e., IL-1β, IL-2, IL-4, IL-5, IL-6, IL-10, IL-12, IL-13, IL-17, GM-CSF, IFN-γ) or neither the “immunogenic” nor the “non-immunogenic” vector lots induced any significant increases in their release (i.e., IL-7, IL-8, G-CSF, MCP-1) (Fig. S2), suggesting a degree of specificity in the AAV-mediated cytokine response.

To investigate cytokine responses at an earlier time point, pDCs were stimulated with the “immunogenic” AAV8 vector lot A-HEK-1. We observed a significant increase in the concentration of TNF-α in the supernatant already at 2 h after pDC stimulation. This response pattern could be confirmed in three experiments, each performed with the cells of one individual donor (Fig. S3).

To investigate whether a higher dose of a “non-immunogenic” AAV8 vector lot was able to trigger an immune response, we stimulated pDCs with the technically maximum applicable MOI (1:4.61 × 10^6^ vg). Interestingly, neither reactive cell proliferation nor cytokine responses were detected upon stimulation with this increased titre (Fig. S4).

Contrary to our hypothesis, these results demonstrate that innate immune responses to AAV in pDCs were lot-specific and not related to a specific production system or manufacturer/purification method.

### The immune response to AAV stimulation in pDCs is not influenced by differences in capsid/vg ratio

**Figure 2:**
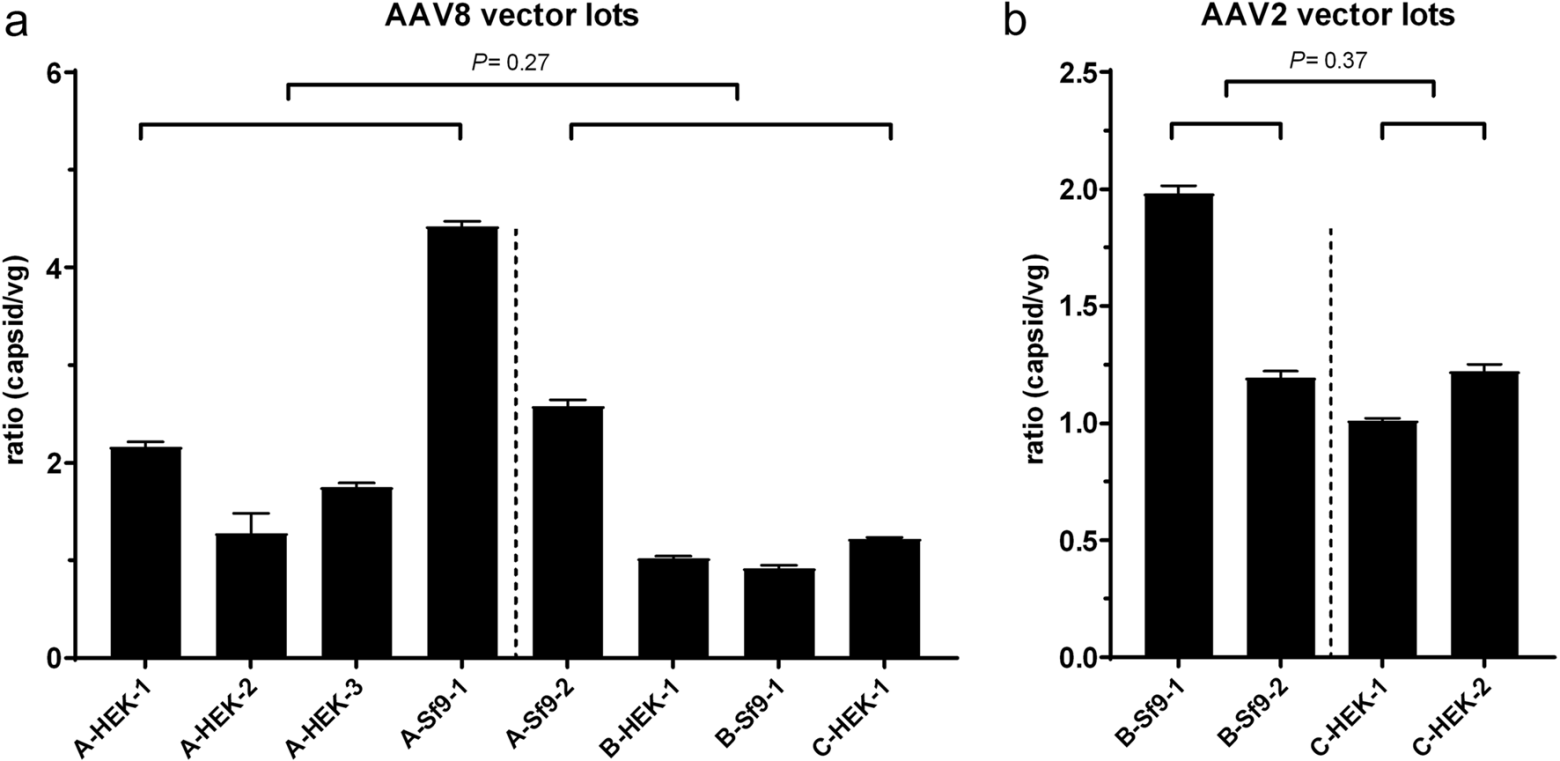
Comparison of capsid/vg ratios between different AAV8-CMV-eGFP and AAV2-CMV-eGFP vector lots. The capsid/vg ratios derive from absorbance measurements (ELISA) and ddPCR results of (**a**) eight AAV8 vector lots and (**b**) four AAV2 vector lots. Dashed lines separate “immunogenic” from “non-immunogenic” AAV vector lots. Bars indicate means and standard deviations of replicates. Statistical significance was determined using unpaired Student t-test.

Preclinical studies have shown that differences in the numbers of full and empty vector particles in AAV vector suspensions can influence an immune response^18^. In order to assess whether the differences in immunogenic properties between the analysed vector lots were due to differences in the ratio of full and empty vector particles, we determined the titre of vector capsids in all AAV lots by AAV titration ELISA and calculated the capsid/vg ratio. Interestingly, large differences in the capsid/vg ratio were found between all AAV lots (Fig. 2). Approximately two times more capsids than vg (2:1) were found in AAV8-CMV-eGFP lots A-HEK-1, A-HEK-3, A-Sf9-2 and AAV2-CMV-eGFP B-Sf9-1; and four times more (4:1) in AAV8 lot A-Sf9-1. A 1:1 ratio was observed in the rest of the lots. However, no significant differences between capsid/vg ratios of “immunogenic” and “non-immunogenic” AAV vector lots were found in AAV8 (*P* = 0.27) nor in AAV2 (*P* = 0.37) vector lots (Fig. 2). This suggests that the differences in the immunogenic properties of the analysed AAV vector lots were also not related to differences in the ratio of full and empty vector particles.

### Recognition of “immunogenic” AAV8 vector lots by TLR9

**Figure 3:**
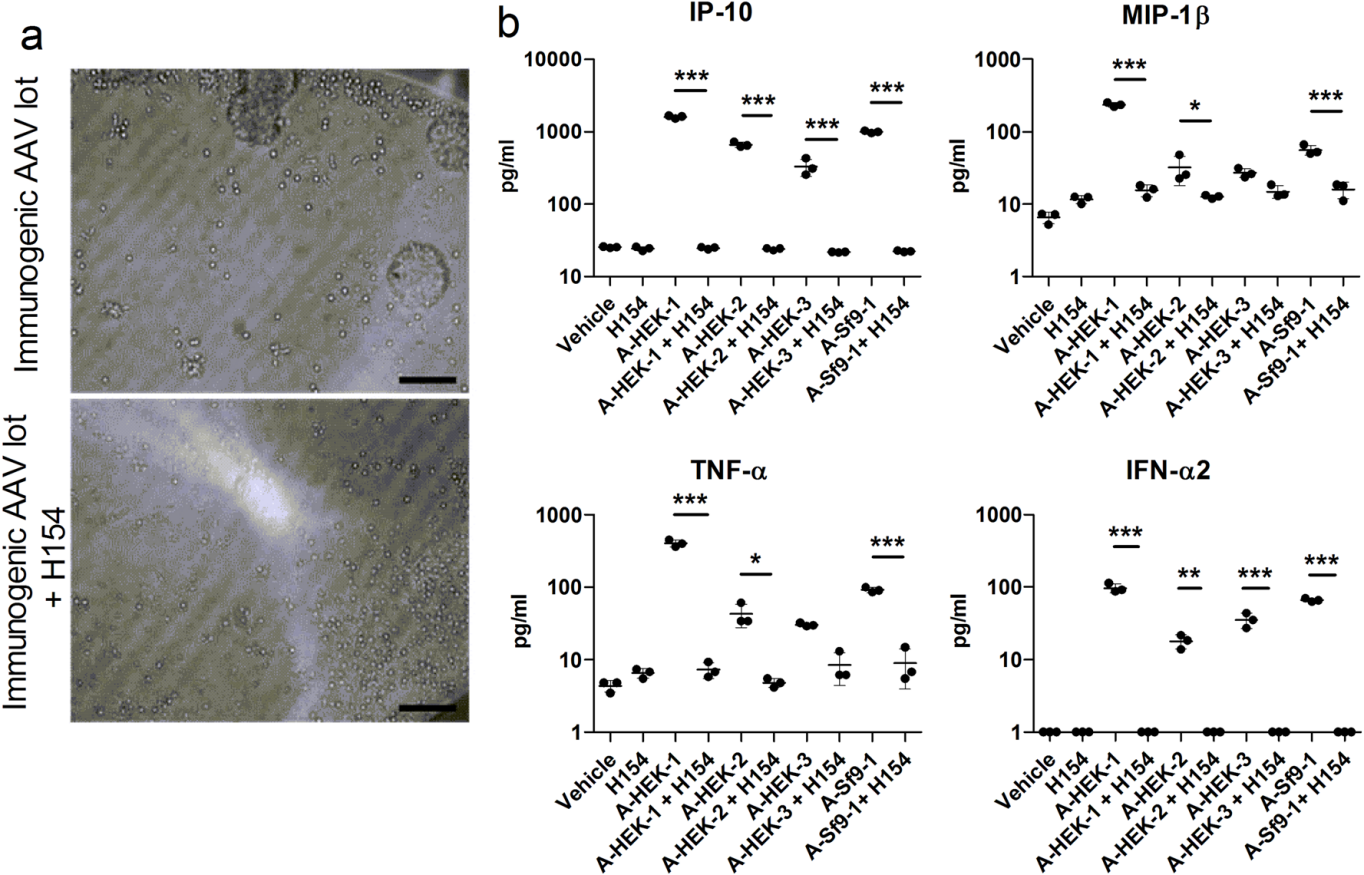
Recognition of “immunogenic” AAV8-CMV-eGFP vector lots by pDCs is TLR9 dependent. Human pDCs were treated with the TLR9 antagonist H154 (50 μM) followed by stimulation with “immunogenic” AAV8 vector lots (MOI: 1:1 × 10^6^ vg) for 18 h. (**a**) Representative bright field images of purified pDCs treated with “immunogenic” AAV vector lots (*upper image*) or “immunogenic” AAV vector lots and H154 (*lower image*). Scale bar is 100 μm. (**b**) Cytokine release of IP-10, MIP-1β, TNF-α and IFN-α2 by stimulated pDCs. Since in the IFN-α2 measurements some values fell below the assay range, the constant 1 was added to all measured IFN-α2 values for presentation in a semi-logarithmic plot. Shown are means and standard deviations. Statistical significance was determined using one-way ANOVA and Holm-Sidak’s post hoc analysis. *P* values: ≤ 0.05: *; ≤ 0.01: **; ≤ 0.001: ***.

It has been shown that the innate immune recognition of AAV by murine and human pDCs is mediated by DNA sensing TLR9^9^. Accordingly, we evaluated whether TLR9 was also involved in the recognition of our “immunogenic” AAV8 vector lots. To this end, pDCs were seeded as described and cultured with the TLR9 antagonist H154 (50 µM) followed by stimulation with the “immunogenic” AAV8 vector lots A-HEK-1, A-HEK-2, A-HEK-3 and A-Sf9-1 (MOI: 1:1 × 10^6^ vg). After 18 h, no evidence of cell proliferation was observed (Fig. 3a) and a significant reduction in the release of IP-10, MIP-1β, TNF-α and IFN-α, was measured (Fig. 3b). This indicates that immune responses to “immunogenic” AAV8 vector lots in human pDCs are mediated by the TLR9 signalling.

### DNase treatment reduces immune responses of “immunogenic” AAV8 and AAV2 vectors lots

While “immunogenic” AAV8 vectors elicited a TLR9-dependent immune response in pDCs, no such responses were induced by the “non-immunogenic” lots. TLR9 is activated in response to DNA, in particular DNA containing unmethylated CpG motifs^9^. Importantly, our ddPCR measurements had confirmed that the same viral DNA components were present in both “immunogenic” and “non-immunogenic” vector lots. Collectively, this suggested that unpackaged/free DNA in the viral suspension rather than the intra-viral DNA could be the causative agent of the observed immune responses to “immunogenic” AAV vector lots. Hence, if residual free DNA was present in the viral suspension of the “immunogenic” AAV8 vector lots, the innate immune response should be attenuated by DNase. To test this hypothesis, “immunogenic” AAV8 and AAV2 vector lots were pre-treated with DNase I (100 µg/ml) for 30 min prior to AAV stimulation. To exclude non-specific effects of DNase treatment on the pDCs or the AAV particles, in the AAV only stimulations the vectors were subjected to DNase-mock treatment prior to pDC stimulation. In these mock treatments without DNase, the AAV particles were incubated at 37 °C in identical DNase digestion medium for the same period of time as the DNase-treated AAVs. Then, pDCs were seeded and stimulated with the DNase pre-treated or mock-treated “immunogenic” AAV vector lots (MOI: 1:1 × 10^6^ vg). pDCs which were incubated with DNase only or sham-treated with vehicle served as controls.

**Figure 4:**
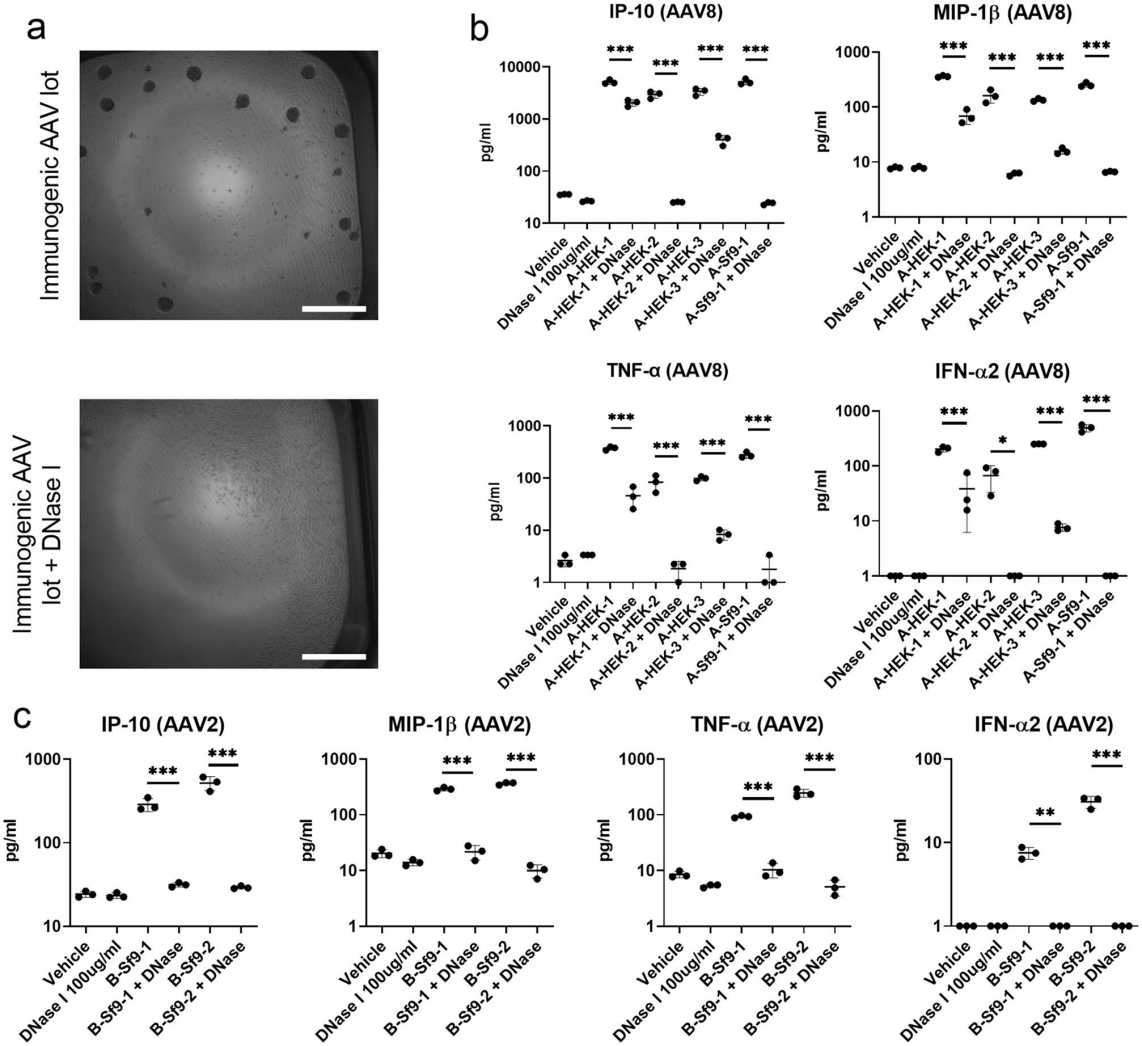
DNase pre-treatment reduces immune responses induced by “immunogenic” AAV8-CMV-eGFP and AAV2-CMV-eGFP vector lots. Human pDCs were stimulated with DNase-treated “immunogenic” AAV8 vector lots for 18 h (MOI: 1:1 × 10^6^ vg). (**a**) Representative bright field images of purified pDCs stimulated with “immunogenic” AAV vector lots (*upper image*) and “immunogenic” AAV vector lots pre-treated with 100 μg/ml of DNase I (*lower image*). Scale bar is 500 μm. (**b**) Cytokine release of IP-10, MIP-1β, TNF-α and IFN-α2 by AAV8-stimulated pDCs. (**c**) Cytokine release by AAV2-stimulated pDCs. Since in the IFN-α2 measurements (**b** and **c**) and TNF-α measurement (**b**) some values fell below the assay range, the constant 1 was added to all measured IFN-α2 and TNF-α values for presentation in a semi-logarithmic plot. Shown are means and standard deviations. Statistical significance was determined using one-way ANOVA and Holm–Sidak’s post hoc analysis. *P* values: ≤ 0.05: *; ≤ 0.01: **; ≤ 0.001: ***.

Interestingly, DNase treatment of the “immunogenic” AAV8 and AAV2 vector lots decreased reactive cell proliferation (Fig. 4a) and either completely abolished (AAV8 lots A-HEK-2 and A-Sf9-1; AAV2 lots B-Sf9-1 and B-Sf9-2) or significantly reduced (AAV8 lots A-HEK-1 and A-HEK-3) the release of IP-10, MIP-1β, TNF-α and IFN-α after AAV stimulation (Fig. 4b,c).

To confirm that the observed DNase effect was actually caused by digestion of extra-viral DNA and not by an effect of the DNase on the integrity of the vector, we investigated whether DNase treatment of AAV vector lots affected the transduction capacity of AAV. Since AAV application did not result in any detectable pDC transduction, HEK cells were used for these assays as HEK cells are a well-known cell model to analyse transduction efficiency upon AAV transduction^19^. We stimulated HEK293T cells with DNase-treated and sham-treated AAV8 and AAV2 vector lots (MOI: 1:8 × 10^4^ vg) and evaluated the transduction efficiency by fluorescence microscopy 3 days after vector application. As described^20^, AAV2 vectors showed a higher transduction potency compared to AAV8 vectors. Importantly, moreover, pre-treatment with DNase neither reduced the transduction efficiency in the HEK-derived nor in Sf9-cell derived AAV8 and AAV2 vector lots (Fig. S5a,b). These results provide further evidence that the DNase effect on the immunogenic properties of the vector lots is due to degradation of the extra-viral DNA.

Although the TLR9 antagonist H154 essentially abolished the release of pro-inflammatory cytokines for all four immunogenic AAV8 lots (Fig. 3), DNase pre-treatment could also eliminate the pro-inflammatory cytokine response for the AAV8 lots A-HEK-2 and A-Sf9-1, while it did not entirely remove it for AAV8 lots A-HEK-1 and A-HEK-3 (Fig. 4). To test whether an increased DNase treatment time could abrogate this cytokine response, we repeated the stimulation experiments of pDCs following a tenfold increase in DNase incubation time of the respective lots (A-HEK-1 and A-HEK-3). We observed that after DNase treatment of the vectors for 5 h, the mean cytokine concentrations in the supernatant of the AAV-stimulated cells further decreased being no longer significantly different from that of vehicle control. This response pattern could be confirmed in three experiments, each performed with the cells of one individual donor (Fig. S6). This suggests that the central cause of the TLR9-dependent pro-inflammatory immune response in pDCs is indeed extra-viral DNA.

**Figure 5:**
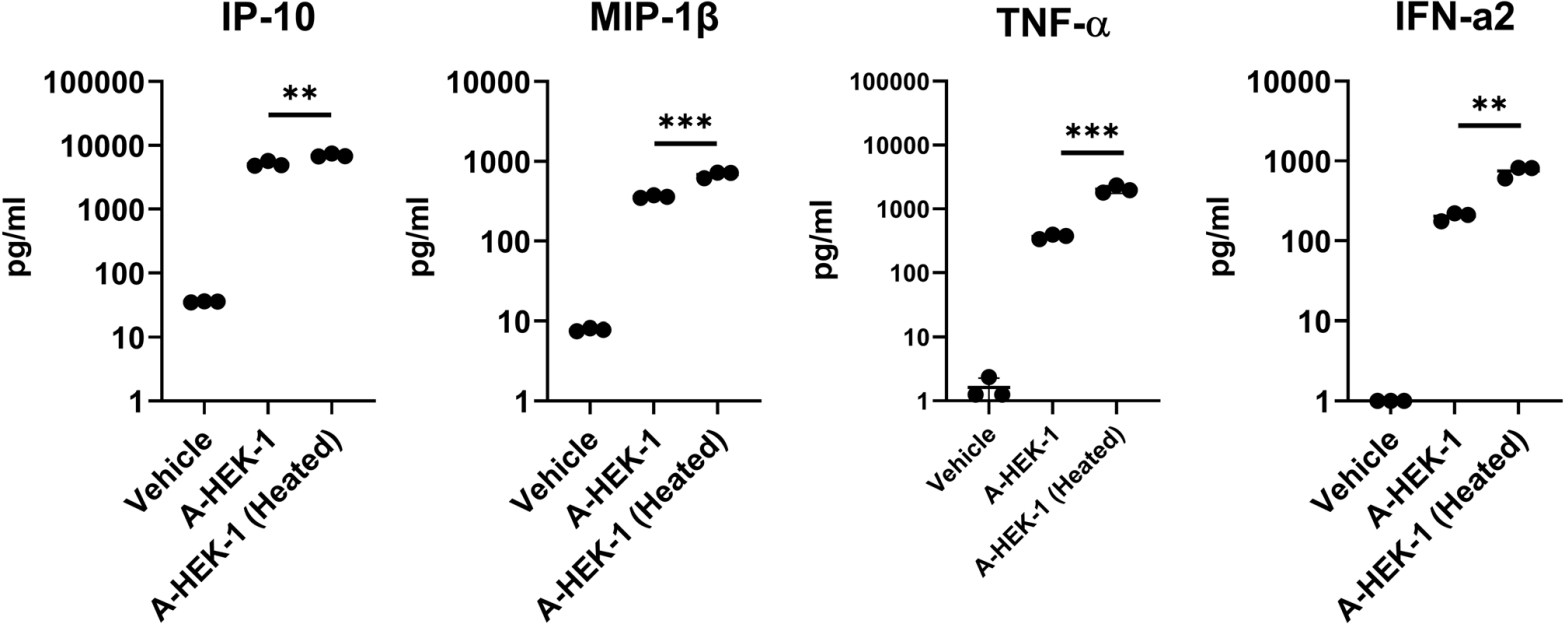
Release of intra-viral DNA by heat-treatment of vectors enhances pro-inflammatory cytokine responses in pDCs. Cytokine release of IP-10, MIP-1β, TNF-α and IFN-α2 by pDCs 18 h after stimulation with heat-treated AAV8 lot A-HEK-1 (MOI: 1:1 × 10^6^ vg). Horizontal lines indicate means and standard deviations. Statistically significant differences between cytokine responses induced by heat-treated and untreated vectors were determined by using Student t-test. *P* value: ≤ 0.01: **; ≤ 0.001: ***.

In order to test whether release of intra-viral DNA increases the immunstimulatory activity of AAV vectors, we deliberately opened the viral capsids of the “immunogenic” AAV8 lot A-HEK-1 by heat-treatment at 95 °C for 10 min. The heat-treated vector lot was then used to stimulate pDCs. Although there were no differences in the reactive cell proliferation upon AAV8 lot A-HEK-1 stimulation with and without heat-treatment, there was a significant increase in the release of IP-10, MIP-1β, TNF-α and IFN-α in the heated-treated condition, indicating that the release of encapsulated viral DNA into the vector suspension can contribute to the induction of immune responses (Fig. 5).

Together, these data indicate that AAV vector preparations may contain extra-viral DNA contaminants that can trigger lot-specific immune responses in pDCs. These responses are mediated by TLR9 signalling and can be reduced/abolished by treatment of the vector lots with DNase.

### Explorative analysis of extra-viral DNA contaminants in AAV8 vector preparations

To perform a first exploratory characterisation of the extra-viral DNA components in the AAV vector lots, a representative “immunogenic” (A-HEK-1) and “non-immunogenic” (B-HEK-1) HEK cell-derived vector lot were analysed. These lots were either DNase-treated in pDC medium for 30 min at 37 °C as described above, or were mock-treated and ultra-filtrated using 100 kDa cut-off filtering device, or were only mock-treated (Fig. S7a–g). Consecutive Bioanalyzer LabChip separation and assessment of the purified DNA revealed the presence of comparable amounts of vector DNA (Fig. S7b–g) in treated and mock-treated samples of each lot confirming that neither ultra-filtration nor DNase-treatment influenced the DNA content inside the capsid. Additionally, it showed that only the “immunogenic” (Fig. S7a–d) but not the “non-immunogenic” vector lot (Fig. S7a,e–g) contained additional DNA molecules ranging in size from 100 to 450 bp (Fig. S7c,d and h). Importantly, these additional DNA molecules could be detected in the mock-treated sample and the ultra-filtrated sample but were virtually absent in the DNase-treated sample (Fig. S7a–d). This indicates that these DNA molecules represent extra-viral DNA contaminants that can be degraded by DNase but cannot be removed from the vector suspension by ultra-filtration. The finding that these contaminants could only be detected in the “immunogenic” but not in the “non-immunogenic” vector lot proves that the occurrence of these extra-viral DNA molecules is lot-specific and might additionally suggest that these molecules induce or contribute to the immunostimulatory properties of the vector.

To assess the potential source of the contaminating DNA in the “immunogenic” (A-HEK-1) and “non-immunogenic” (B-HEK-1) lots quantitative (q)PCR analysis of HEK cell DNA, plasmid DNA and AAV vector DNA was performed. Template DNA from DNase-treated, mock-treated and ultra-filtrated and only mock-treated samples of each lot was used for amplification of Alu repeat, nuclear genome multicopy NPIP gene, and mitochondrial 16S rRNA gene sequences (mt16S) of HEK cell origin, for the amplification of an AAV8 inverted terminal repeat amplicon (ITR2; vector and transgene plasmid DNA origin), and for the amplification of an amplicon for the *bla* (ampicillin resistance) gene (Amp; plasmid DNA origin) (Table S4). Comparison of the ratios of the ΔCt values of HEK cell DNA-specific amplicon vs the ITR2 amplicon (i.e. Alu vs. ITR2, NPIP vs. ITR2, and mt16S vs. ITR2) and the plasmid DNA-specific amplicon versus the ITR2 amplicon (Amp vs. ITR2), respectively indicates that the two lots contain negligible amounts of HEK cell nuclear and mitochondrial DNA. In contrast, the analysis revealed a decent amount of plasmid DNA in both the “immunogenic” and the “non-immunogenic” vector lot (about 1/32th and 1/50th, respectively, in terms of copy number relative to AAV DNA), a proportion which is well within the range of values reported for other AAV vectors^12^ (Fig. S7). Nonetheless, there were no obvious differences in the relative proportion of vector DNA to plasmid and HEK cell DNA between the DNase-treated and mock-treated or ultra-filtrated and mock-treated samples of both vector lots (Fig. S8). This may suggest that the non-packaged contaminant DNA is similar in target sequence composition to that of the packaged DNA. However, a limitation of the qPCR analysis is the size of the contaminant DNA (100–450 bp) which will contain a decent fraction of fragments with incomplete target sequence.

## Discussion

AAV vectors are one of the most promising tools in gene therapy. However, accumulating evidence challenges the view that immunogenicity of AAV is negligible^5^. In light of this, it has become increasingly important to better understanding of the mechanisms by which immune responses to AAV occur.

In this study we demonstrate that (1) AAV8 and AAV2 induce lot-specific innate immune responses in human pDCs which are neither specific to the capsid/vg ratio nor the production platform nor the manufacturer/purification method; (2) innate immune responses in pDCs are dependent on TLR9 signalling and can be reduced by pre-treatment with DNase; (3) DNase treatment does not affect the integrity of the vector particle as it does not reduce the transduction rate of AAV8 and AAV2 vector lots in HEK293T cells; and (4) AAV vector lots can comprise extra-viral DNA molecules which are can be removed by treatment of the vector lot with DNase. This suggests that that both HEK- and *Sf9*-cell derived AAV preparations can contain extra-viral DNA impurities that stimulate an innate immune response.

In a recent study, a comparative analysis was carried out using AAV vectors from different host cell species (HEK cells and *Sf9* cells)^11^. The authors found that HEK- and *Sf9*-derived vectors differ in terms of their PTMs and their residual host cell protein impurities across all AAV serotypes and manufacturers they tested^11^. Furthermore, they analysed the cytokine response of primary human fibroblasts to AAV transduction and found that HEK- and *Sf9*-derived vectors may differ in their immunogenic properties. In our study, we also compared these two main production systems using different lots of the same AAV construct from different manufacturers and two different serotypes. However, the vector-induced immune responses we observed in our human pDC model were not specifically related to a given production system, manufacturer or serotype, instead they were lot-specific.

Previous pre-clinical and clinical studies of retinal gene therapy have reported that immune responses to AAV vectors, such as ocular inflammation or immune cell infiltration, may be influenced by differences in capsid/vg ratios^18^ or dose differences^5^. Timmers et al.^18^ showed that removing empty AAV capsids from the viral suspension reduced inflammation and improved viral transduction in a pre-clinical study with non-human primates. Yet, our results showed that elevated capsid/vg ratios were present among both “immunogenic” and “non-immunogenic” AAV vector lots, meaning that a higher number of capsids (empty capsids) in the viral suspension was not responsible for the induction of the vector lot-specific immune responses in our human pDC model. Besides that, we have previously shown that AAV8 induces immune responses in a dose-dependent manner in non-human primates^6,8^. However, increasing the dose of the “non-immunogenic” AAV vectors in this study was not sufficient to trigger an immune response in human pDCs. Therefore, to understand the cause of the differences in the immunogenic properties of the vectors, we investigated the mechanism involved in the recognition of the “immunogenic” AAV lots.

The use of in vitro immunocompetent cell models has allowed scientists to more accurately study the role of TLR pathways in innate immune responses generated by AAV vectors^9,10^. Zhu et al.^9^ first described that pDCs, but not conventional DCs or non-pDCs, release large amounts of type I IFN and pro-inflammatory cytokines in response to AAV stimulation and demonstrated the involvement of the TLR9 pathway in the recognition of AAV8 and AAV2 using mouse pDCs. The authors also observed that AAV2 induced TLR9-dependent immune responses in human pDCs. In our study, with the use of one of the most specific TLR9 antagonists, H154^9,21–24^, we have indirectly shown that not only AAV2, but also AAV8 vectors induce TLR9-dependent innate immune responses in human pDCs, but in a lot-specific manner.

As TLR9 is a DNA receptor, this suggested that the immune responses to “immunogenic” AAV vector lots were induced by DNA components. However, although our ddPCR measurements had confirmed that the same packaged DNA components were present in both “immunogenic” and “non-immunogenic” vector lots, the “non-immunogenic” lots did not trigger immune responses in pDCs. Additionally, DNase treatment either reduced or abolished the immunostimulatory properties of the “immunogenic” AAV vector lots, but did not decrease the transduction potency of these vectors in HEK cells, suggesting that DNase treatment of AAV targeted non-encapsulated extra-viral DNA in the vector suspension but did not affect the integrity of the vector genome within the intact capsid. This was further confirmed by comparative DNA analyses of representative DNase-treated and mock-treated “immunogenic” and “non-immunogenic” vector lots. Collectively, all of these experiments indicate that the immune response to “immunogenic” vector lots in pDCs was not orchestrated by DNA contained in the AAV particles (i.e. vector genome or potential DNA impurities packaged in the AAV capsids) but by accessible extra-viral DNA components contained in the viral suspensions (i.e. DNA outside or unprotected by the viral capsid).

We also observed that when the DNA contained in the viral capsids of an “immunogenic” vector lot was exposed to the cells by heat-treatment of the vector there was an increase the immune response. It is not clear whether the increase in immunostimulatory activity after opening the capsids was due to the single stranded DNA of the vector or due to potential impurities packaged in the AAV capsids such as residual host cell nucleic acids, residual helper DNA sequences, backbone sequence fragments packaged along with the cassette or reverse priming from ITRs resulting in small backbone fragments packaged into the AAV^12,25^.

The exact mechanism of extra-viral DNA uptake by pDCs remains unknown. Responsiveness of pDCs to CpG oligonucleotides demonstrates that pDCs can react to free DNA^26^. This suggests that the immune response in pDCs might be triggered by the uptake of free contaminating DNA contained in the vector suspension. Additionally, it has been shown that pDCs can take up AAV vectors^27^ alternatively suggesting that potential capsid-bound DNA could be transported into the cell during uptake of AAV particles.

The presence of extra-viral DNA contamination in AAV vector preparations could either be an inherent property of the respective vector lot resulting from the production and purification process of the vectors, or it could be due to the release of encapsulated DNA due to inappropriate storage conditions. All vector lots examined in these experiments were obtained during the same time period (5 of the 8 AAV8 vector lots examined, of which two were “immunogenic” (A-HEK-2; A-HEK-3) and three were “non-immunogenic” (A-Sf9-2; B-Sf9-1; B-Sf9-2) were even produced in parallel specifically for this study), the lots from each manufacturer were shipped in the same transport boxes and, upon receipt, all lots were stored side-by-side in the same drawer of a temperature alarm-protected − 80 °C freezer before aliquots of all lots were thawed simultaneously and applied to the pDCs in side-by-side experiments. This strongly suggests that the AAV production and purification process, rather than inappropriate storage, was responsible for the differences in extra-viral DNA content and immunostimulatory properties of these lots.

In the past, clinical grade AAV vector lots produced for gene therapy (alipogene tiparvovec, Glybera) were no longer authorised due to high amount of impurities including potentially immunogenic residual host cell DNA^28^. The removal of DNA impurities from the viral suspensions is usually performed during the production process. Here Benzonase^29^ or DNase^30^ treatment is routinely applied in order to eliminate residual DNA from the final viral suspension. This treatment, depending on the protocol, lasts between 30 min and 3 h and then the enzyme is inactivated by chemicals such as caesium chloride salts^30^. In our experiments four of the five AAV8 lots derived from the Viral Vector Core Facility of the University of Iowa and both AAV2 vector lots from Virovek were found to be immunogenic in pDCs. As described in “Materials and methods” section, the purification process of the vector lots from both of these manufactures involves several purification steps, some of which differ greatly from each other. Thus, the purification process at the Core Facility in Iowa includes Turbonuclease treatment, followed by iodixanol gradient ultracentrifugation, anion exchange column chromatography, filter sterilization and buffer exchange using centrifugal filters. In contrast, Virovek applies Benzonase treatment, ultracentrifugation in CsCl followed by desalting and filter sterilization. The purification process of Vigene, on the other hand, does not comprise DNase treatment, but, as is used by Core Facility in Iowa, includes iodixanol gradient ultracentrifugation. Surprisingly, however, none of the three vector lots derived from Vigene (AAV8: C-HEK-1; AAV2: C-HEK-1 and 2) induced immune responses in pDCs, suggesting that "non-immunogenic" vector lots can also be generated with production processes that do not involve DNase treatment. Overall, the partly similar and partly different purification steps used by the three manufacturers make it difficult to link the immunogenic properties of the vectors observed in pDC to a single step in the manufacturing process.

Clinical trials use good manufacturing practice (GMP)-grade vectors which are subjected to stringent quality controls. None of the vectors from the three manufacturers used in this study are good laboratory practice (GLP) grade and may therefore be of lower purity than clinical grade vectors. Thus, it is important to recognize that GMP vectors used in clinical trials in humans may or may not exhibit the differences we observed in the present study and that the relevance of our findings for clinical use of gene therapies is not clear. However, in our own experience, considerable lot-specific differences in the concentration of contaminating components such as host cell proteins can occur even with GMP-grade vectors and even in GMP-grade vectors there are no uniformly agreed specifications as to what levels are acceptable^5^. Collectively, improving the manufacturing process of AAV vectors is key to avoid the presence of residual impurities in the viral suspensions in order to minimize the potential for unintended immune responses^12,31,32^.

In conclusion, we demonstrated that extra-viral DNA impurities can influence the immunogenic properties of AAV vectors in human pDCs. Further studies are required to investigate the implications of these findings for the safety of AAV-mediated gene therapy in animal models or human patients.

## Materials and methods

### AAV vector lots

The experimental AAV vectors used in this study comprised eight AAV8 vector lots and four AAV2 vector lots all containing the identical DNA sequence of the CMV promoter and the identical transgene for eGFP (Table 1). They were derived from two different production systems: transient transfection of human HEK cells and live baculovirus infection of *Sf9* insect cells; and obtained from three different manufacturers: (A) Viral Vector Core Facility of the University of Iowa (Iowa, USA), (B) Virovek (CA, USA) and (C) Vigene Biosciences (MD, USA). The same plasmid (pFB-CMV-eGFP, 7122 bp) was used by University of Iowa and Virovek to manufacture their AAV8 lots (lots A-HEK-1, A-HEK-2, A-HEK-3, A-Sf9-1, A-Sf9-2, B-HEK-1, B-Sf9-1). A similar plasmid was used by Vigene to produce AAV8 lot C-HEK-1 (pAV-CMV-eGFP, 5030 bp).

### Vector production and purification

#### Viral vector core facility of the University of Iowa

Cell Culture: HEK 293FT cells were maintained adherently in Dulbecco’s modified Eagle’s medium (DMEM) (Gibco) supplemented with 10% fetal bovine serum (FBS), 1% penicillin, and 1% streptomycin at 37 °C and 5% CO_2_. Sf9 insect cells were maintained in suspension in Sf-900 II SFM medium (Gibco) supplemented with 7.5% FBS, 1% penicillin, 1% streptomycin and 0.02% gentamicin or in in serum‐free ESF921 medium (Expression Systems Inc.) supplemented with 1% penicillin G, and 1% streptomycin at ~ 120 rpm and 27 °C.

Virus Production: For AAV8 production in HEK cells, the eGFP expression plasmid was co-transfected with a plasmid containing the viral rep and cap genes (rep/cap plasmid) and a plasmid containing the adenovirus-helper genes (helper plasmid) into HEK 239FT cells. After 72 h the cells were harvested and AAV was purified as described below. For AAV8 production in Sf9 cells, cells were co-infected at an MOI of 1:0.5 each with two baculovirus expression vectors: a baculovirus vector carrying the eGFP transgene and another baculovirus vector encoding the rep and cap genes. The helper elements were already present in the Baculovirus genome. After 72 h the cells were harvested and AAV was purified as described below.

Purification of all AAV preparations: Cells were pelleted by centrifugation. Then, pellets were lysed, treated with a detergent and Turbonuclease (100 U/ml) and then they were spun down again. Afterwards, AAV8 vectors were purified first using an iodixanol gradient followed by a Mustang Q anion exchange column chromatography and 0.22 um filtration. Finally, the purified virus samples were concentrated and buffer exchanged using Ultracel centrifugal filters (Millipore) with a 100,000 MW cutoff into a target volume of DPBS containing final concentration of 180 mM NaCl, and 0.001% Pluronic F68 (Sigma). Physical titres (in vg/ml) were determined by qPCR against a standard plasmid curve.

#### Virovek

For AAV production in Sf9 cells, cells were co-infected with a baculovirus expression vector encoding the eGFP transgene and another baculovirus vector encoding the rep and cap genes. Cells were harvested and lysed and treated with Benzoase (8 U/ml). AAV vectors were purified using ultracentrifugation in CsCl. Following desalting and filter sterilization, qPCR was performed to determine the viral titre. Additionally, SDS-PAGE analysis served to verify the purity of the AAV vectors. For AAV production in HEK cells, the eGFP expression plasmid was co-transfected with the rep/cap plasmid and the helper plasmid) into the attached HEK cells. Then cells were collected and lysed. Afterwards the AAV were treated and purified in the same way as the Sf9-cell produced vectors.

#### Vigene

AAV production in HEK was performed by triple transfection of the eGFP expression plasmid, the rep/cap plasmid and the helper plasmid in the attached cell line. No treatment with DNase/Benzonase was performed. Afterwards, AAV samples were purified by iodixanol gradient ultracentrifugation.

### Droplet digital PCR genome titre assay

ddPCR was carried out in order to precisely re-quantify the vector genome (vg) titres of the purchased AAV vectors in a side-by-side measurement. The reaction mixtures were assembled with 10 µl ddPCR Supermix (Bio-Rad, Hercules, CA, USA), TaqMan primers and probes (Applied Biosystems, Foster, CA, USA) (final concentrations of 10 µM), and template (5 µl) in a final volume of 20 µl. In order to ensure consistent results, reaction mixtures were prepared with 2 different primers/probes: CMV assay (Forward: 5′-GCACCAAAATCAACGGGACT-3′; Reverse: 5′-CTCCCACCGTACACGCCTAC-3′; Probe: 5′-6FAM-AATGTCGTAACAACTCCG-MGB-3′) and eGFP assay (Forward: 5′-GGAGCGCACCATCTTCTTCA-3′; Reverse: 5′-CAGGGTGTCGCCCTCGA-3; Probe: 5′-6FAM-CTACAAGACCCGCGCCGAGGTG-MGB-3′). Each reaction was loaded into the sample well of an eight-well disposable cartridge (Bio-Rad) along with droplet generation oil (Bio-Rad), and droplets were generated in a droplet generator (Bio-Rad). Droplets were transferred to a 96-well PCR plate, sealed, and amplified to the end point (95 °C for 10 min, followed by 40 cycles of 94 °C for 30 s, 56 °C for 1 min, and 72 °C for 15 s followed by a final 98 °C heat treatment for 10 min). The PCR plate was subsequently scanned on a Q × 100 droplet reader (Bio-Rad) and the data were analysed with QuantaSoft software (Bio-Rad).

### Titration of capsid particles by ELISA

The capsid particle titres of the AAV vector lots were determined using three separate dilution series from each sample using an AAV8 titration ELISA kit or AAV2 titration ELISA kit (Progen Biotechnik GmbH, Heidelberg, Germany). The AAV vector lots were diluted with ready-to-use sample buffer so that they could be measured within the range of the ELISA and transferred to the ELISA plate according to the manufacturer’s instructions. Briefly, 100 µl of serial dilutions of standard and specimens were pipetted into the wells and incubated for 1 h at 37 °C. Then, biotin conjugate was added to the wells and incubated for 1 h at 37 °C followed by the streptavidin conjugate (1 h, 37 °C) and the substrate solution (15 min at room temperature). Absorbance was quantified using M200 NanoQuant spectrophotometer (Tecan, Männedorf, Switzerland) at 450 nm (650 nm correction wavelength).

### Isolation of human pDCs

Human pDCs were purified from buffy coats from healthy young donors which were obtained from the Center for Clinical Transfusion Medicine (Tübingen, Germany). The study was approved by the Ethics Committee of the Medical Faculty and University Hospital of Tuebingen, Germany (no. 139/2020BO2), informed consent was obtained from all subjects and all methods were carried out in accordance with relevant guidelines and regulations.

To purify pDCs PBMCs were isolated using Ficoll density gradient centrifugation (Ficoll-Paque; GE Healthcare, Uppsala, Sweden). pDCs were purified from PBMCs by MACS by negative selection using biotin-conjugated antibodies and anti-biotin microbeads (Miltenyi Biotec, Bergisch Gladbach, Germany). Purity assessment was performed by flow cytometry. Cells were stained using PE Mouse Anti-Human CD123 and BV421 Mouse Anti-Human BDCA-2 (CD303) antibodies (Becton Dickinson Bioscience, NJ, USA). Measurement was performed on a FACSCantoTM II (Becton Dickinson Bioscience) and data were evaluated with FlowJo software. The purified pDCs were seeded at 12,500 cells/well into 384-well plates (Corning, New York, NY, USA) in 50 µl of medium containing RPMI (Sigma-Aldrich, San Luis, MO, USA) with 10% human serum (Sigma-Aldrich), 1% GlutaMAX (Gibco, Thermo Fisher Scientific, MA, USA), 1% MEM Non-Essential Amino Acids (Gibco), 1% Sodium Pyruvate (Gibco) and 1% Penicillin/Streptomycin (P/S; Gibco). Immediately after seeding, cells were stimulated as described below and were incubated for 18 h at 37 °C. Cell proliferation and eGFP expression was assessed using an Axioplan2 imaging fluorescent microscope (Zeiss, Oberkochen, Germany) after 18 h. Supernatants were collected and stored at − 80 °C until being assayed.

### Stimulation of human pDCs

In side-by-side experiments using the cells from individual donors, pDCs were stimulated with different lots of AAV8-CMV-eGFP and AAV2-CMV-eGFP (Table 1) at a multiplicity of infection (MOI) of 1:1 × 10^6^ vg. Type A CpG oligodeoxynucleotide (ODN 2216; Invivogen, San Diego, CA, USA) at 0.77 µM was used as positive stimulation control.

In order to block TLR9, 50 µM of phosphorothioate-stabilized ODN H154 (5′-CCTCAAGCTTGAGGGG-3′; Biomers.net, Ulm, Germany) was applied to the pDCs followed by stimulation with AAV lots.

To remove potential extra-viral DNA components in viral suspensions, AAV vector lots were pre-treated with 100 µg/ml of deoxyribonuclease I (DNase I, Stemcell Technologies, Vancouver, Canada) in buffer with calcium and magnesium (PAN-Biotech GmbH, Aidenbach, Germany) for 30 min at 37 °C, prior to application to the pDCs.

### Detection of cytokines by multiplex assay

Initial screening of cytokine concentrations in the supernatant of the stimulated pDCs was performed using Bio-Plex Pro Human Cytokine 17-Plex Panel (Bio-Rad), complemented by a Bio-Plex Pro Human Cytokine IP-10 (Bio-Rad) and Bio-Plex Pro Human Cytokine IFN-a2 (Bio-Rad) resulting in a total of 19 cytokine targets (IP-10, MIP-1β, TNF-α, IFN-α, IL-1β, IL-2, IL-4, IL-5, IL-6, IL-7, IL-8, IL-10, IL-12, IL-13, IL-17, G-CSF, GM-CSF, IFN-γ and MCP-1). Consecutive analyses were performed using a combination of Bio-Plex Pro Human Cytokine IP-10, Bio-Plex Pro Human Cytokine MIP-1α, Bio-Plex Pro Human Cytokine TNF-α and Bio-Plex Pro Human Cytokine IFN-a2 sets (Bio-Rad).

Multiplex assay was performed on a Luminex 200 system (Bio-Rad) in accordance with the manufacturer’s instructions. Standards were analysed in duplicates and each sample in triplicates. Washing was performed between each step using a HydroFlex microplate equipped with a magnetic plate carrier (Tecan). Analysis of the data was performed using Bio-Plex Manager (Bio-Rad).

### Transduction of HEK293T cells

Human embryonic kidney (HEK) 293 T cell line (CRL-3216™) was purchased from ATCC (Manassas, VA, USA) and cultured in 96-well plates (Corning) with DMEM + GlutaMAX (Gibco) supplemented with 10% heat-inactivated foetal bovine serum (hiFBS, Gibco) and 1% P/S (Gibco). When the cells reached 70–80% confluence of the plate, they were stimulated with different lots of AAV8-CMV-eGFP (MOI: 1:8 × 10^4^ vg) and AAV2-CMV-eGFP (MOI: 1:1.6 × 10^4^ vg) or with AAV lots pre-treated with DNase I (100 µg/ml; Stemcell). Expression of eGFP was monitored under the Axioplan2 imaging fluorescent microscope (Zeiss).

### DNA analysis of the AAV preparations

Aliquots of AAV preparations were thawed on ice and treated with 100 µg/ml DNase I in pDC medium for 30 min 37 °C. EDTA was added to a final concentration of 15 mM and the DNase I heat inactivated for 15 min at 72 °C. Samples were then lysed with SDS and Tween-20 (end concentration 0.3% and 4.5%, respectively) und digested with Proteinase K (final concentration 1 mg/ml) for 2.5 h at 55 °C. Subsequently, samples were extracted twice with an equal volume of phenol–chloroform-isoamylalcohol (25:24:1) and finally purified and desalted on a Microspin G-25 spin column (Cytiva, Marlborough, MA, USA). Two mock-treated AAV samples (i.e. without DNase I) from the same lot were processed in parallel. Prior to the lysis step, one of mock-treated samples was diluted to 1.8 ml with Dulbecco’s phosphate buffered saline (with sodium chloride adjusted to 180 mM) + 0.001% Pluronic und ultrafiltrated on a Centricon YM-100 spin cartridge (Millipore, Bedford, MA, USA) for 12 min at 2000 g in a fixed angle rotor. Retenate volumes were 60–70 µl.

Total DNA from AAV preparations was separated on a Agilent Bioanalyzer 2100 instrument (Agilent, Waldbronn, Germany) using the High Sensitivity DNA Kit. 5 ng of ΦX-174 DNA digested with HaeIII was run alongside on the labchip as an additional control for size and amounts of DNA.

Quantitative PCR (qPCR) was done using SYPR Green chemistry (QuantiTect SYBR Green PCR Kit, Qiagen, Hilden, Germany) on an ABI 7500 instrument (ThermoFisher/Applied Biosystems, Weiterstadt, Germany). 5 µl of a 1:50 dilution of template DNA was used for amplification of the Alu, NPIP, and mt16S amplicons (Table S4), and 5 µl of a 1:5000 dilution for the ITR2 and Amp amplicons (Table S4). Reaction volumes were 20 µl and triplicate reactions were done for each template/assay. Mean Ct of the triplicates were used to calculate ΔCt values.

### Statistical analysis

Statistical analysis was carried out using GraphPad Prism (GraphPad Software, San Diego, CA, USA) and JMP (SAS Institute Inc, Cary, NC, USA). To determine lot-specific differences in cytokine responses determined in independent experiments, linear mixed-effects models, fit by expected mean squares (EML), were used to assess the statistically significance of the fixed effects Lot and Replicate (nested in Lot) in explaining variations in the measured concentrations of the different cytokines. Because of large differences in the scale of the cytokine concentrations a Yeo-Johnson power transform was applied before fitting the model. To account for inter-individual differences donors were included as random effect. Prior to utilizing the results of the models, the normal distribution of the model residuals was confirmed visually, and the homoscedasticity of the variances of the residual was ensured using the Brown–Forsythe test and reported in case of violations. Post-hoc testing using Dunnet’s test was used to compare the least-squares means of the estimated cytokine concentrations against the control (Vehicle). In order to assess the significance among pairs of conditions, a one-way analysis of variance (ANOVA), followed by Holm–Sidak’s *post* hoc test was performed. Student’s t test was used when only two conditions were tested. Prior to these analyses, the normal distribution of the data was confirmed by the Shapiro–Wilk test.

## Supplementary Information

Supplementary Information.

## Data Availability

The datasets generated and analyzed during the current study are available from the corresponding author on reasonable request.
