# Supplementary material for: Extra-viral DNA in adeno-associated viral vector preparations induces TLR9-dependent innate immune responses in human plasmacytoid dendritic cells
Source: Sci Rep. 2023 Feb 2;13:1890. doi: 10.1038/s41598-023-28830-7 (PMC9894911; doi:10.1038/s41598-023-28830-7)
Supplement: Supplementary file 1 — Supplementary Information. [file 41598_2023_28830_MOESM1_ESM.pdf]

Supplementary Information

Figure S1

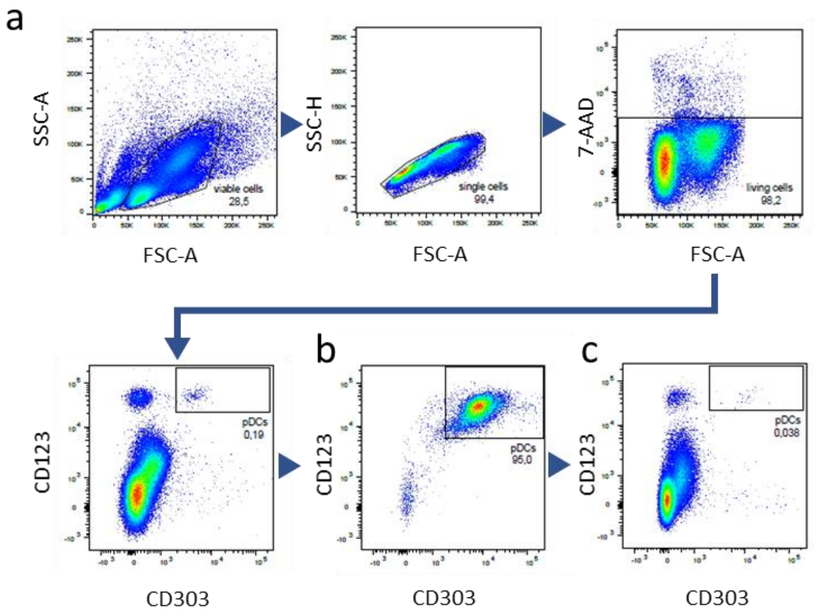

Supplementary Figure 1. Determination of the purity of plasmacytoid dendritic cells (pDCs) after PBMC isolation and enrichment by magnetic cell sorting (MACS). pDCs were stained with fluorescent antibodies and analysed by flow cytometry. pDCs were gated as single, 7-AAD negative living cells that were positive for CD123 and CD303. (a) Gating strategy for pDCs shown by the example of the complete PBMC fraction before MACS enrichment (total PBMCs). (b) Percentage of pDCs after MACS (pDCs). Enriched living pDCs had a purity of over 90%. (c) Percentage of pDCs in the remaining PBMC fraction after `removal´ of pDCs by MACS (PBMC-pDCs).

Figure S2

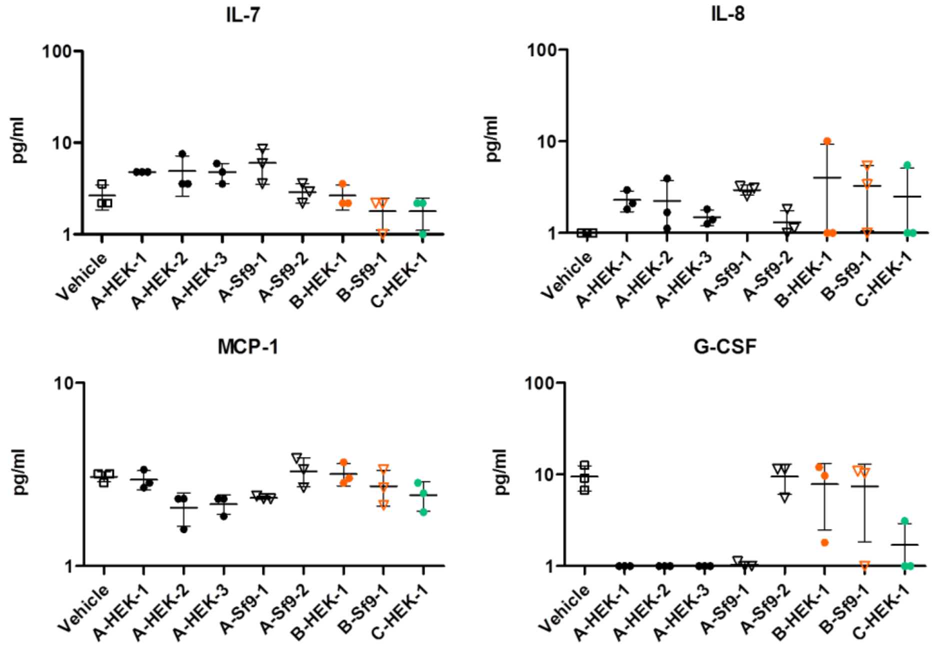

Supplementary Figure 2. Cytokine release of IL-7, IL-8, MCP-1 and G-CSF of pDCs stimulated with different lots of AAV8-CMV-eGFP at 18 hours. Since in the IL-7, IL-8 and G-CSF measurements some values fell below the assay range, the constant 1 was added to all measured values for presentation in a semi-logarithmic plot. Shown are means and standard deviations.

Figure S3

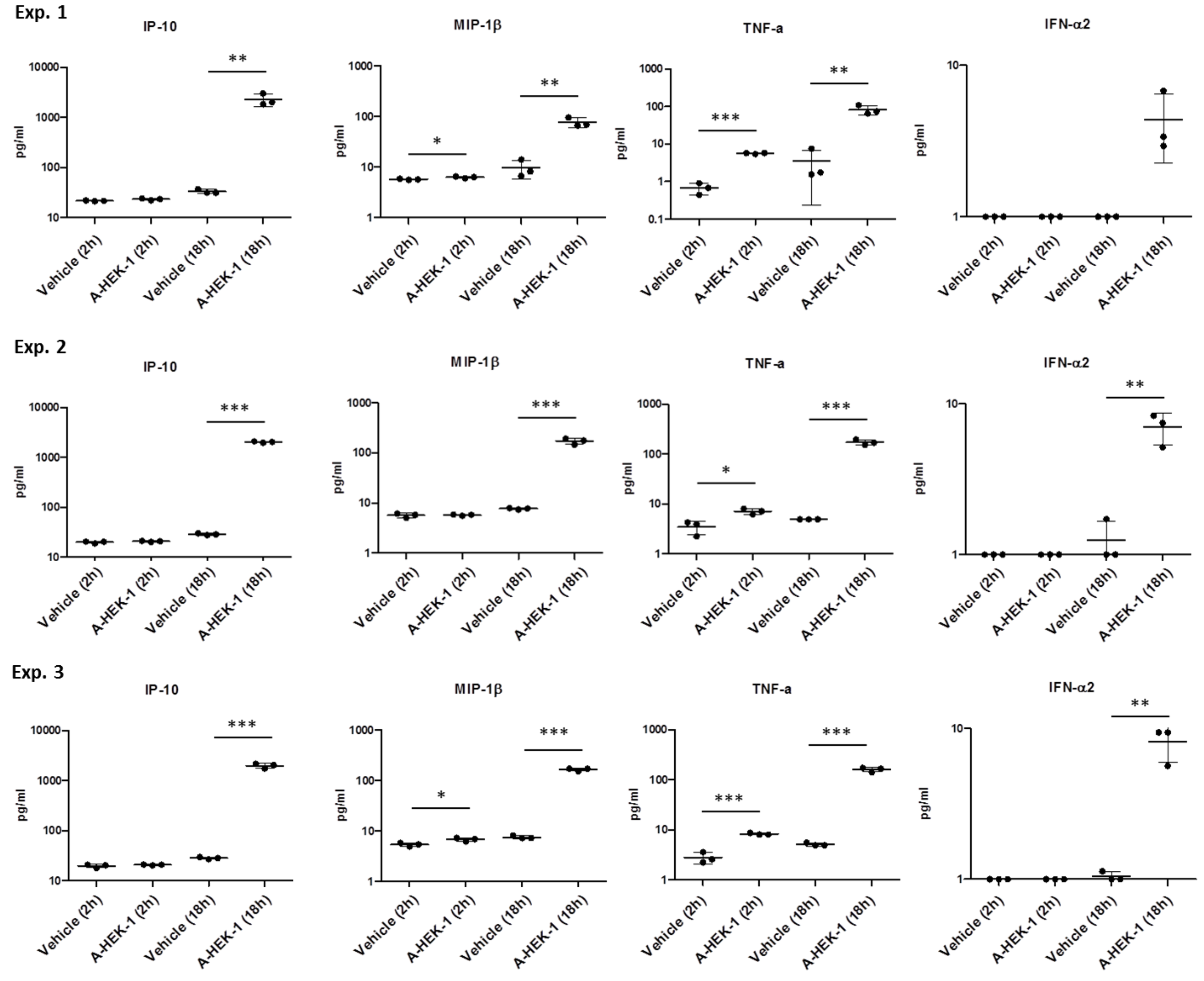

Supplementary Figure 3. Stimulation with an "immunogenic" AAV8 vector lot induces early immune responses in human pDCs. Primary human pDCs were stimulated with AAV8-CMV-eGFP (Lot A-HEK-1; MOI: 1:1x10^6 vg) for 2h and for 18h. Incubation of pDCs in vehicle containing medium (vehicle) served as control. Cytokine concentrations of IP-10, MIP-1β, TNF-α and IFN-α2 in the supernatant. Each panel shows the results of one experiment performed with the cells of an individual donor (Exp1 – 3). Since in the IFN-α2 measurement some values fell below the assay range, the constant 1 was added to all measured values for presentation in a semi-logarithmic plot. Shown are means and standard deviations. Statistically significant differences between AAV-stimulated pDCs and the respective controls were determined using unpaired Student t-test. P-values: ≤0.05: *; ≤0.01: **; ≤0.001: ***.

Figure S4

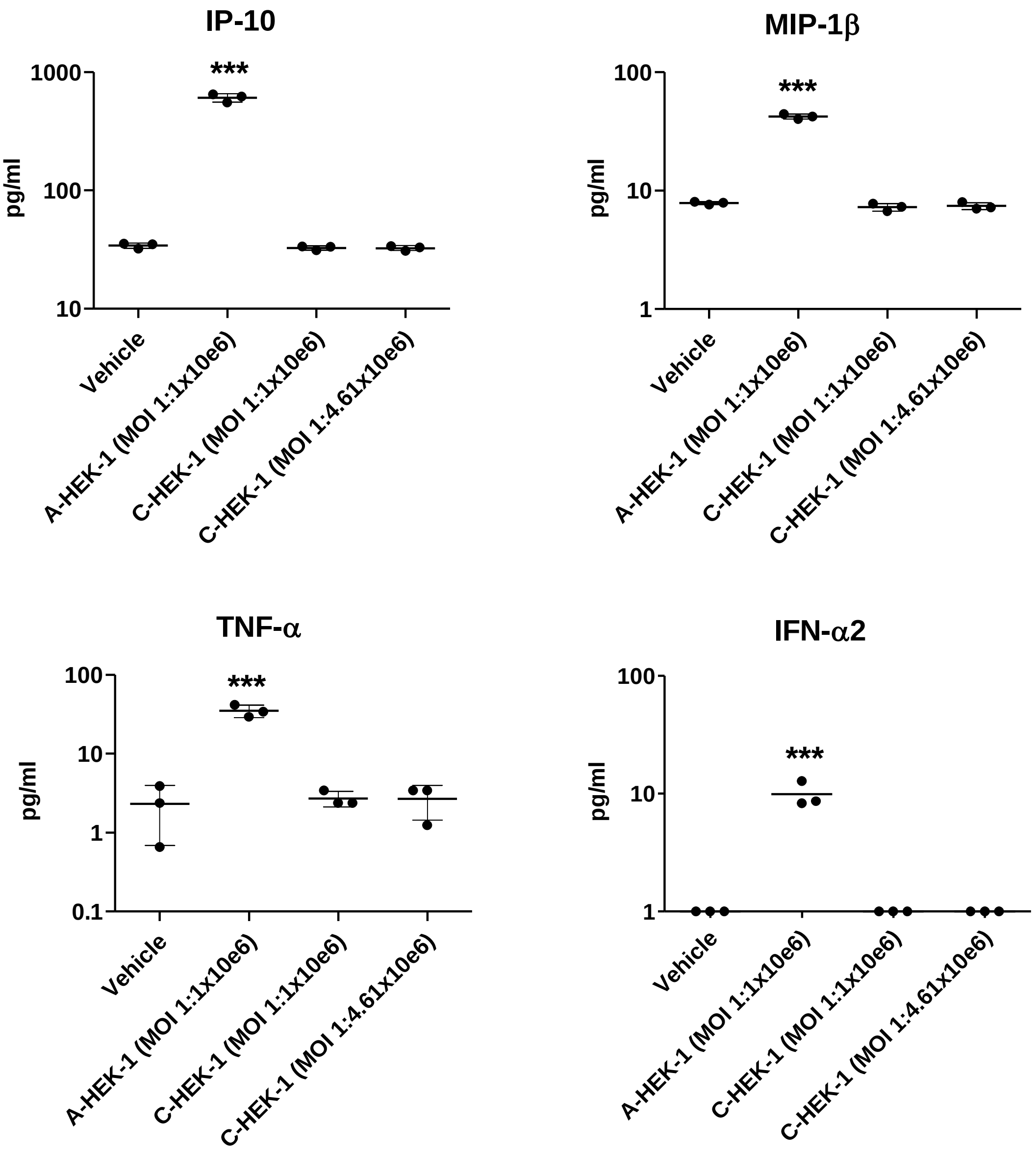

Supplementary Figure 4 . Increasing the MOI of a "non-immunogenic" AAV vector does not induce immune responses in human pDCs. Primary human pDCs were stimulated with the "non-immunogenic" vector lot C-HEK-1 at an MOI of $1:1x10^6$ vg and at the technically maximum applicable MOI ($1:4.61x10^6$ vg) for 18h. Incubation of pDCs with the "immunogenic" vector lot A-HEK-1 served as positive stimulation control. Cytokine concentrations of IP-10, MIP-1β, TNF-α and IFN-α2 in the supernatant. Since in the IFN-α2 measurement some values fell below the assay range, the constant 1 was added to all measured values for presentation in a semi-logarithmic plot. Shown are means and standard deviations. Statistical significance was determined using one-way ANOVA and Holm-Sidak's post hoc analysis. Asterisks indicate significant differences in comparison to the vehicle control. P-values: ≤0.001: ***.

Figure S5

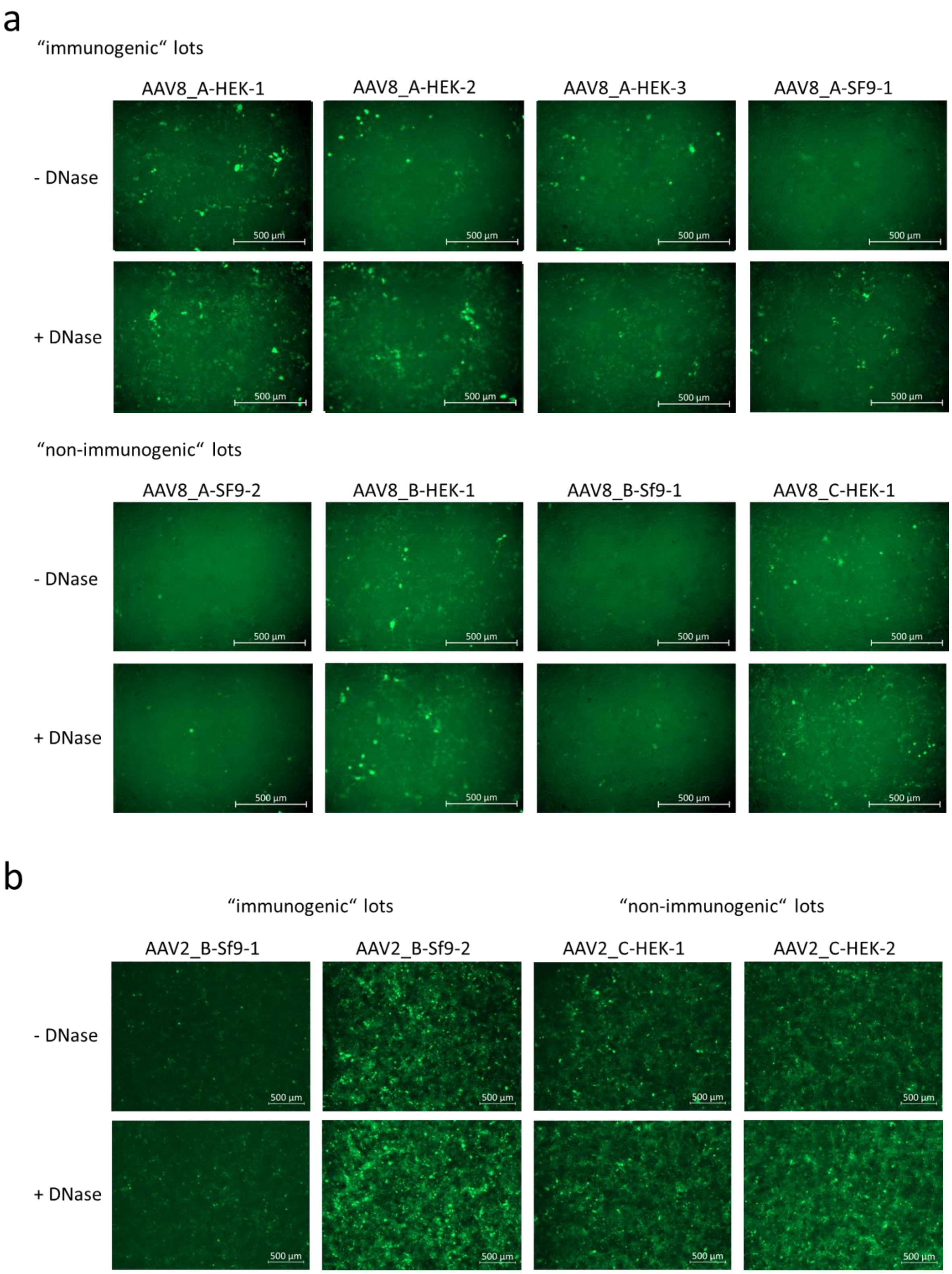

Supplementary Figure 5. DNase pre-treatment does not decrease the transduction efficiency of AAV8-CMV-eGFP and AAV2-CMV-eGFP vector lots in HEK293T cells. HEK293T cells were stimulated with DNase-treated or sham-treated vector lots for 3 days (MOI: 1: 1:8x10^4 vg). eGFP expression as determined by fluorescence microscopy after stimulation with “immunogenic” and “non-immunogenic” AAV8-CMV-eGFP (a) and AAV2-CMV-eGFP vector lots (b).

Figure S6

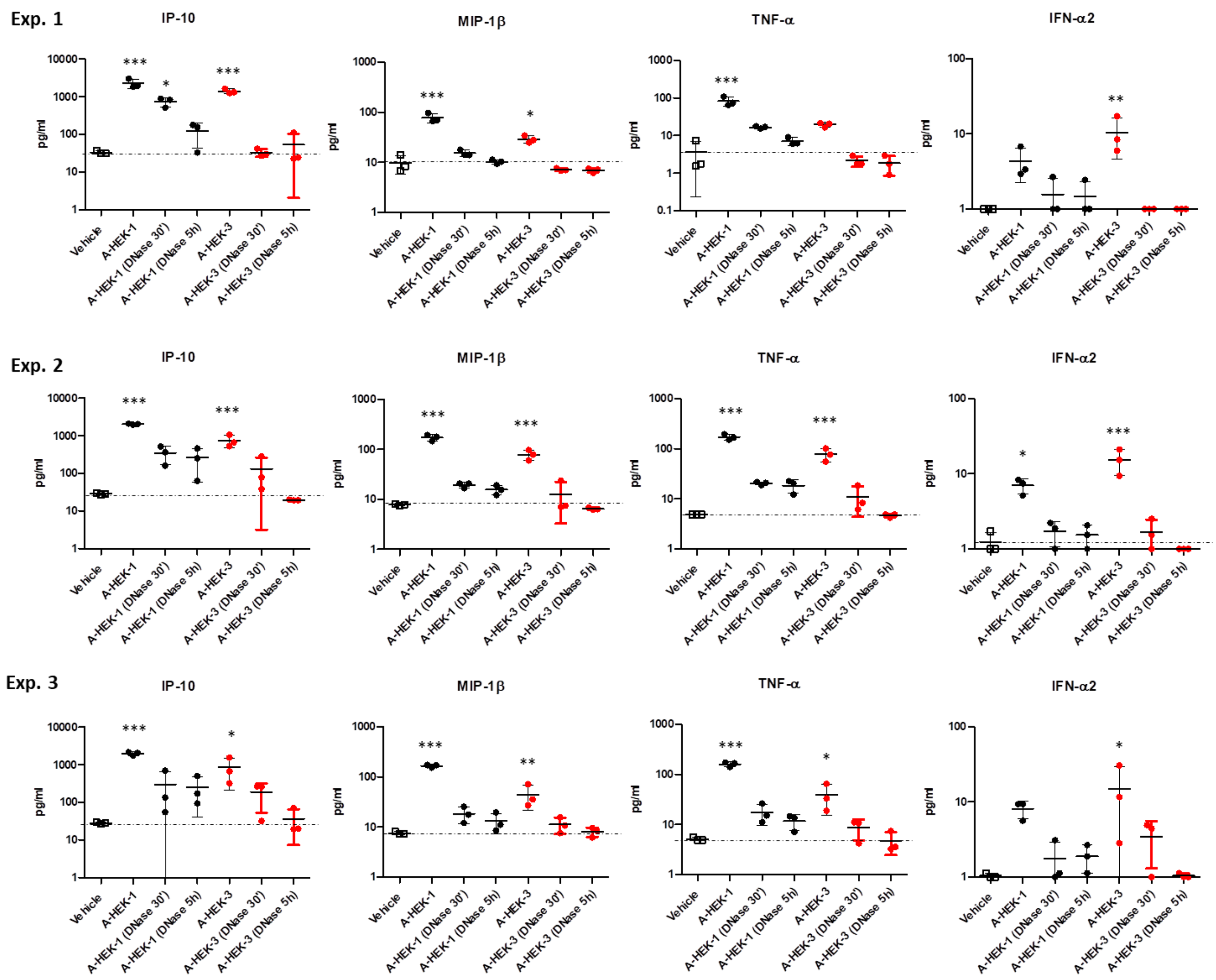

Supplementary Figure 6. Increased DNase incubation time of "immunogenic" vector lots further reduces immune responses in human pDCs. "Immunogenic" vector lots AAV8 lots A-HEK-1 and A-HEK-3 were pre-treated with DNase for 30 min and 5h. Then, pDCs were stimulated with the DNase-treated vector lots for 18h (MOI: 1:1x106 vg). Incubation of pDCs in vehicle containing medium (vehicle) served as control. Cytokine concentrations of IP-10, MIP-1β, TNF-α and IFN-α2 in the supernatant. Each panel shows the results of one experiment performed with the cells of an individual donor (Exp1 – 3). Since in the IFN-α2 measurement some values fell below the assay range, the constant 1 was added to all measured values for presentation in a semi-logarithmic plot. Shown are means and standard deviations. Statistical significance was determined using one-way ANOVA and Holm-Sidak's post hoc analysis. Asterisks indicate significant differences in comparison to the vehicle control. P-values: ≤0.05: *; ≤0.01: **; ≤0.001: ***.

Figure S7

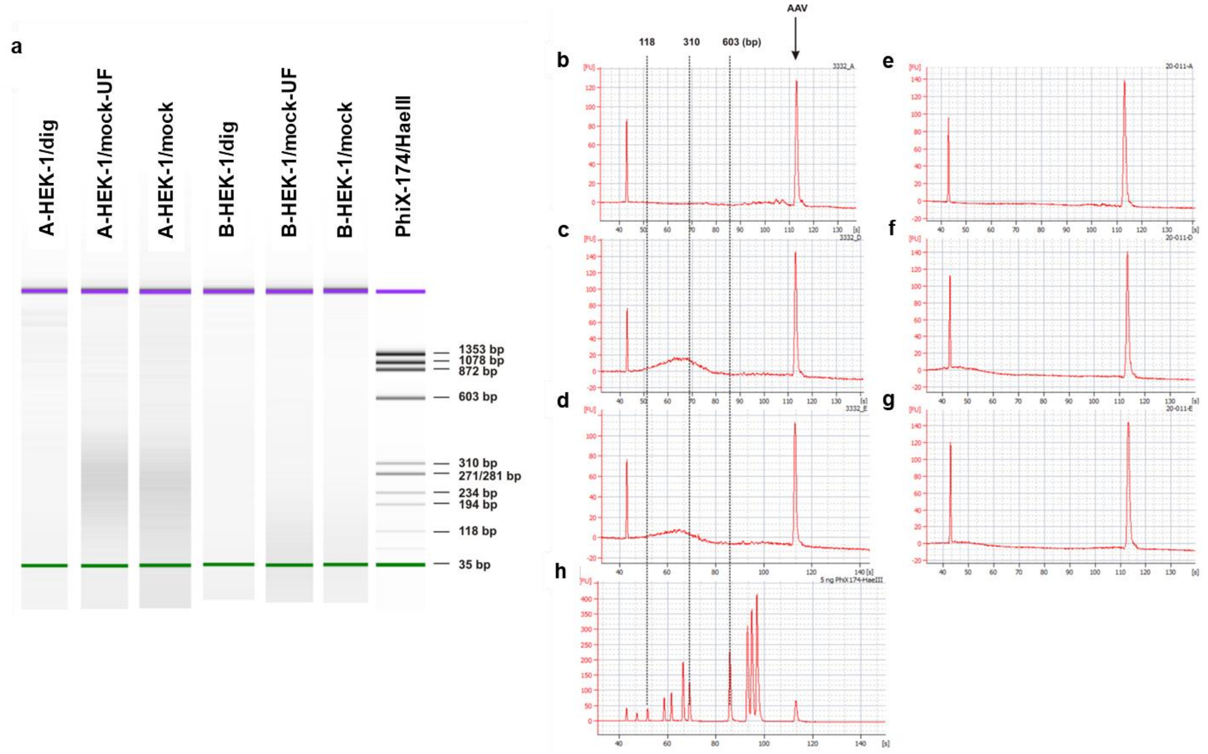

Supplementary Figure 7. Bioanalyzer 2100 LabChip separation of DNA from DNase I and mock digested AAV preparations (a) Virtual gel picture of DNA isolated from "immunogenic" (A-HEK-1) and "non-immunogenic" (B-HEK-1) AAV8 preparations after DNAse I treatment (dig) or mock treatment of samples with or without additional ultrafiltration (mock-UF and mock, respectively). Rightmost lane shows separation of the PhiX-174/HaeIII DNA marker. (b-d) Corresponding electropherograms of DNase I treated (b) or mock treated samples with (c) or without ultrafiltration (d) of the "immunogenic" A-HEK-1 AAV8 preparation. The arrow in (b) points to the peak of the AAV vector DNA. (e-g) Corresponding electropherograms of DNase I treated (e) or mock treated samples with (f) or without ultrafiltration (g) of the "non-immunogenic" B-HEK-1 AAV8 lot preparation. (h) Corresponding electropherogram of the PhiX-174/HaeIII DNA marker. Note, that AAV DNA (mostly single-stranded) co-migrates with the upper marker band with the used High Sensitivity DNA Kit.

Figure S8

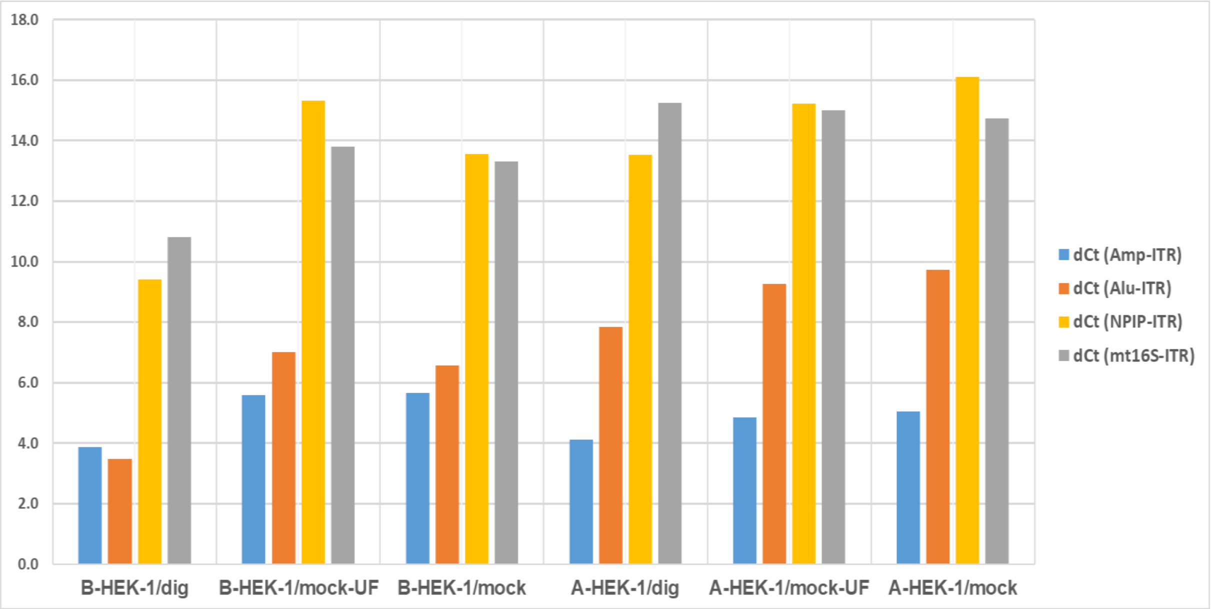

Supplementary Figure 8. qPCR analysis of DNase I and mock digested AAV preparations Bars represent ΔCt values obtained for Amp vs ITR2 (blue), Alu vs ITR2 (orange), NPIP vs ITR2 (yellow), and mt16S vs ITR2 (grey) amplicons for "non-immunogenic" (B-HEK-1) and "immunogenic" (A-HEK-1) AAV8 preparations after DNAse I treatment (dig) or mock treatment of samples with or without additional ultrafiltration (mock-UF and mock, respectively). Note that ΔCt values where calculated from the mean of triplicate Ct values obtained with 1:50 diluted (Alu, NPIP, and mt16S amplicons) or 1:5000 diluted (ITR2 and Amp amplicons) DNA preparations. Few reactions which did not reach fluorescence threshold of 0.05 (NPIP and mt16S amplicons) were attributed with a threshold Ct of 38. ΔCt values (y) correspond to a 2^{y} fold excess of the ITR target sequence compared to the Amp target sequence, and a $2^{y+6.64}$ fold excess of the ITR target sequence compared to the Alu, NPIP, and mt16S target sequences in the samples under optimal amplication conditions.

Supplementary Table 1: Cytokine concentrations in the supernatant of human pDCs stimulated with AAV8-CMV-eGFP and AAV2-CMV-eGFP vector lots measured in three to four independent experiments (Exp.1 – 4) each performed with the cells of one individual donor.

AAV8											
			Vehicle	A-HEK-1	A-HEK-2	A-HEK-3	A-Sf9-1	A-Sf9-2	B-HEK-1	B-Sf9-1	C-HEK-1
IFN-α	Exp. 1	Median	0.91	11.54	2.51			0.91		0.91	1.96
		IQR	0.53	7.92	0.36			1.1		1.38	1.77
	Exp. 2	Median	0	35.85	26.95	73.3	253.35	0	0	0	0
		IQR	0	16.01	2.81	31.96	128.57	0	0	0	0
	Exp. 3	Median	0	315.27	0	52.58	2.34	0	0	0	0
		IQR	0	122.89	0.5	20.91	12.31	0	0	0	0
	Exp. 4	Median	0	90.66	17.4	33.97	64.31				
		IQR	0	25.57	7.82	16.53	7.08				
IP-10	Exp. 1	Median	25.09	688.32	32.35			24.76		26.73	24.42
		IQR	17.13	674.74	32.11			1		5.76	3.98
	Exp. 2	Median	20.68	2236.79	1766.54	1178.34	2935.8	26	26.97	24.74	20.68
		IQR	3.45	366.83	157.29	692.58	580.27	87.51	4.28	1.84	1.46
	Exp. 3	Median	24.55	2049.47	550.71	2218.16	597.66	26.33	26.19	26.33	22.17
		IQR	1.66	235.7	260.76	643.33	1537.85	1.63	0.52	2.52	4.82
	Exp. 4	Median	25.52	1623.66	644.09	310.66	989.51				
		IQR	0.97	146.83	105.81	172.99	69.51				
MIP-1β	Exp. 1	Median	59.73	76.92	82.93			67.87		70.58	70.45
		IQR	5.54	18.92	8.41			5.85		2.58	11.43
	Exp. 2	Median	6.87	248.42	156.15	128.56	321.47	7.72	8.66	10.13	7.07
		IQR	1.09	65.55	37.79	50.04	55.79	2.55	2.08	14.3	2.4
	Exp. 3	Median	11.08	605.24	46.28	105.8	29.39	12.66	7.43	6.67	7.01
		IQR	6.54	39.99	10.63	40.34	29.99	2.17	2.03	9.41	3.99
	Exp. 4	Median	7.24	233.69	25.52	26.28	52.21				
		IQR	2.14	33.21	25.49	7.84	16.15				
TNF-α	Exp. 1	Median	11.97	30.06	19.45			13.08		13.56	14.36
		IQR	1.12	10.42	4.28			2.39		1.6	0.95
	Exp. 2	Median	3.22	187.5	84.08	81.07	352.63	4.59	3.91	7.46	2.75
		IQR	1.62	90.9	28.44	33.87	130.92	2.25	1.62	13.14	2.29
	Exp. 3	Median	6.14	1436.69	31.61	139.23	26.15	4.83	4.83	4.83	3.82
		IQR	2.29	318.13	9.69	17.09	34.78	1.98	1.67	7.19	1.37
	Exp. 4	Median	4.83	396.2	34.17	29.9	89.82				
		IQR	1.35	87.95	26.57	3.15	14.7				

AAV2							
			Vehicle	B-Sf9-1	B-Sf9-2	C-HEK-1	C-HEK-2
IFN-α	Exp. 1	Median	0	40.84	59.17	0	0
		IQR	0	19.94	48.5	0	0
	Exp. 2	Median	0	0	0	0	0
		IQR	0	0	1.19	0	0
	Exp. 3	Median	0	6.47	32.26	0	0
		IQR	0	2.43	8.9	0	0
IP-10	Exp. 1	Median	35.7	3012.7	3184.15	42.01	31.69
		IQR	1.21	455.55	2715.06	20.26	2.06
	Exp. 2	Median	34.1	322.49	488.24	49.43	40.88
		IQR	5.42	404.3	656.57	19.44	26.52
	Exp. 3	Median	23.94	263	536.05	26.38	29.33
		IQR	4.04	94.14	197.27	1.25	1.67
MIP-1β	Exp. 1	Median	7.77	65.08	92.45	7.23	5.8
		IQR	0.67	30.56	21.14	0.29	1.01
	Exp. 2	Median	285.59	477.89	522.76	389.6	345.7
		IQR	64.71	39.73	91.64	56.87	40.86
	Exp. 3	Median	18.61	288.81	376.23	13.63	13.96
		IQR	5.91	42.06	37.4	2.65	1.47
TNF-α	Exp. 1	Median	1.25	49.85	89.88	0	1.25
		IQR	1.11	14.21	20.98	2.36	1.25
	Exp. 2	Median	42.33	143.7	196.38	69.83	65.68
		IQR	10.49	32.86	76.51	25.19	23.65
	Exp. 3	Median	8.03	92.97	234.05	4.88	5.53
		IQR	2.14	9.35	78.14	1.3	3.21

In the labels of the individual vector lots the three the three manufacturers are represented by the letters A, B, C; HEK-cell derived and Sf9-cell derived vectors are indicated by "HEK and "Sf9" and corresponding lots of the same manufacturer and the same production system are numbered “1, 2, 3”. IQR, interquartile range.

Supplementary Table 2: Results of the linear-mixed effect models for the Yeo-Johnson transformed cytokine concentrations of the independent experiments.

	Cytokine	Effect	*F*-statistics	*p*-value
**AAV8**	IFN-a<br>Yeo-Johnson λ = 0.0	N = 87, $r^2_{adj.}$ = .69, p < .0001, Brown-Forsythe: $F(1, 85)$ = 27.01, p < .0001		
		Condition	F(8, 57) = 22.79	< .0001
		Replicates[Condition]	F(18, 57) = 0.08	1.0000
	IP-10<br>Yeo-Johnson λ = -0.6	N = 87, $r^2_{adj.}$ = .88, p < .0001, Brown-Forsythe: $F(1, 85)$ = 1.42, p = .2365		
		Condition	F(8, 57) = 68.50	< .0001
		Replicates[Condition]	F(18, 57) = 0.27	.9982
	MIP-1b<br>Yeo-Johnson λ = -0.1	N = 87, $r^2_{adj.}$ = .62, p < .0001, Brown-Forsythe: $F(1, 85)$ = 4.49, p = 0.0369		
		Condition	F(8, 57) = 19.01	< .0001
		Replicates[Condition]	F(18, 57) = 0.09	1.0000
	TNF-a<br>Yeo-Johnson λ = -0.3	N = 87, $r^2_{adj.}$ = .74, p < .0001, Brown-Forsythe: $F(1, 85)$ = 1.56, p = .2150		
		Condition	F(8, 57) = 31.39	< .0001
		Replicates[Condition]	F(18,57) = 0.17	.9999
**AAV2**	Cytokine	Effect	*F*-statistics	*p*-value
	IFN-a2<br>Yeo-Johnson λ = -0.92	N = 45, $r^2_{adj.}$ = .62, p < .0001, Brown-Forsythe: $F(1, 43)$ = 3.50, p < .0682		
		Condition	F(4, 28) = 17.27	< .0001
		Replicates[Condition]	F(10, 28) = 0.09	.9998
	IP-10<br>Yeo-Johnson λ = -0.4	N = 45, $r^2_{adj.}$ = .94, p < .0001, Brown-Forsythe: $F(1, 43)$ = 3.80, p = .0579		
		Condition	F(4, 28) = 179,86	< .0001
		Replicates[Condition]	F(10, 28) = 0.33	.9670
	MIP-1b<br>Yeo-Johnson λ = 0.12	N = 45, $r^2_{adj.}$ = .85, p < .0001, Brown-Forsythe: $F(1, 43)$ = 4.86, p = .0329		
		Condition	F(4, 28) = 21.55	< .0001
		Replicates[Condition]	F(10, 28) = 0.02	1.0000
	TNF-a<br>Yeo-Johnson λ = 0.18	N = 45, $r^2_{adj.}$ = .87, p < .0001, Brown-Forsythe: $F(1, 43)$ = 4.12, p = .0487		
		Condition	F(4, 28) = 50,85	< .0001
		Replicates[Condition]	F(10, 28) = 0.16	.9978

Supplementary Table 3: Results of the Posthoc Dunnet's Test (vs. Control = Vehicle)

	**AAV8**							
Vehicle vs. Cytokine	A-HEK-1	A-HEK-2	A-HEK-3	A-Sf9-1	A-Sf9-2	B-HEK-1	B-Sf9-1	C-HEK-1
	p-value							
IFN-α	< .0001	.0015	< .0001	< .0001	.9993	1.0000	.9997	.9976
IP-10	< .0001	< .0001	< .0001	< .0001	.8167	1.0000	.9995	.9938
MIP-1β	< .0001	.0001	< .0001	< .0001	.9998	.8576	1.0000	.9989
TNF-α	< .0001	< .0001	< .0001	< .0001	.9973	.9678	.8123	1.0000

	**AAV2**			
Vehicle vs. Cytokine	B-Sf9-1	B-Sf9-2	C-HEK-1	C-HEK-2
	p-value			
IFN-α	.0001	<.0001	1.0000	1.0000
IP-10	<.0001	<.0001	0.1577	0.3470
MIP-1β	<.0001	<.0001	1.0000	0.9939
TNF-α	<.0001	<.0001	0.9984	1.0000

Supplementary Table 4: qPCR assays

Assay	Target	Forward Primer (5'-3')	Reverse Primer (5'-3')	Size	Ref
ITR2	AAV inverted terminal repeat	GGAACCCCTAGTGATGGAGTT	CGGCCTCAGTGAGCGA	62	1
Amp	*bla* (Amp^r) gene	CGCGCCACATAGCAGAACTT	CGCCCCGAAGAACGTTT	56	2
Alu	Human Alu repeat	CAGGAGATCGAGACCATCC	TGCCTCAGCCTCCCAAG	117+	3
NPIP	Human multicopy NPIP gene	TGGCATGAATAGTGTGGGATTT	GGGGAAGTCTTCGTCTTCACTC	84	
Mt16S	Human mitochondrial 16S rRNA gene	GCCTTCCCCCGTAAATGATA	TTATGCGATTACCGGGCTCT	97	4

1. Shmidt AA, Egorova TV. PCR-Based Analytical Methods for Quantification and Quality Control of Recombinant Adeno-Associated Viral Vector Preparations. Pharmaceuticals (Basel). 2021 Dec 24;15(1):23. doi: 10.3390/ph15010023. PMID: 35056080.
2. Hauck B, Murphy SL, Smith PH, Qu G, Liu X, Zelenaia O, Mingozzi F, Sommer JM, High KA, Wright JF. Undetectable transcription of cap in a clinical AAV vector: implications for preformed capsid in immune responses. Mol Ther. 2009 Jan;17(1):144-52. doi: 10.1038/mt.2008.227. PMID: 18941440
3. Nicklas JA, Buel E. Development of an Alu-based, real-time PCR method for quantitation of human DNA in forensic samples. J Forensic Sci. 2003 Sep;48(5):936-44. PMID: 14535658.
4. Venegas V, Wang J, Dimmock D, Wong LJ. Real-time quantitative PCR analysis of mitochondrial DNA content. Curr Protoc Hum Genet. 2011 Jan; Chapter 19:Unit 19.7. doi: 10.1002/0471142905.hg1907s68. PMID: 2123487
